# Mathematical modelling of the effects of public health educational campaigns on the transmission dynamics of *Taenia solium* Taeniasis

**DOI:** 10.1016/j.parepi.2026.e00528

**Published:** 2026-08-04

**Authors:** Gideon Eustace Rwabona, Abdoelneser Degoot, Silas Mirau, Sayoki Mfinanga, Verdiana Grace Masanja

**Affiliations:** a School of Computational and Communication Sciences and Engineering, P. Box 447, Arusha, Tanzania; b Department of Mathematics and Statistics, Mbeya University of Science and Technology, P. Box 31, Mbeya, Tanzania; c African Institute for Mathematical Sciences (AIMS-Rwanda), Kigali, Rwanda; d National Institute for Medical Research (NIMR), Dar es Salaam, Tanzania; e Kampala International University in Tanzania (KIUT), Dar es Salaam, Tanzania

**Keywords:** *Taenia solium*, Effective reproduction number, Global stability, Numerical analysis, Public health education, Zoonotic transmission

## Abstract

*Taenia solium* Taeniasis is a zoonotic intestinal infection that poses a significant threat to public health and imposes a substantial economic burden on developing regions, particularly in Sub-Saharan Africa. This study formulates and rigorously analyses a deterministic mathematical model, comprised of a system of non-linear ordinary differential equations, to investigate the transmission dynamics of the parasite under the influence of public health education campaigns. Using the next-generation matrix method, we derive the effective reproduction number, $R_{ed}$, as the fundamental threshold for disease persistence. Stability analysis demonstrates that the disease-free equilibrium is globally asymptotically stable when $R_{ed}<1$, ensuring disease eradication, whereas the infection persists and reaches a stable endemic equilibrium when $R_{ed}>1$. Numerical simulations are provided to validate the analytical findings and to explore the parameter space associated with behavioural modification. The results indicate that public health education is a highly effective control measure, capable of significantly reducing prevalence and potentially eliminating *T. solium* Taeniasis in endemic settings.

## Highlights

• Modelled *T. solium* Taeniasis disease dynamics incorporating public health education and knowledge loss.
• Identified host transmission rates as the primary drivers of infection persistence.
• Shown that linking porcine management to human health is vital for disease control.
• Recommended continuous educational campaigns to counteract knowledge attrition.

## 1 Introduction

*Taenia solium* Taeniasis refers to infection of humans with the adult stage of the pork tapeworm, *T. solium*. It is an intestinal parasitic infection that occurs when the adult worm inhabits the human small intestine (Mwasunda, 2022). The infection is acquired when humans consume raw or undercooked pork contaminated with the larval stage of the parasite (O’Neal et al., 2014). The causative agent, *T. solium*, has a complex life cycle involving two distinct hosts: humans, who serve as the sole definitive hosts, and pigs, which act as intermediate hosts (Mwasunda, 2022).

In pigs, the larval stages develop after ingestion of *T. solium* eggs from a contaminated environment (Winskill et al., 2017). Infected humans contaminate the environment by shedding *T. solium* eggs in their feces. These eggs are highly resilient and can survive in the environment for several days to months (Omeragić et al., 2023). Pigs become infected when they ingest vegetation, feed, or water contaminated with these eggs. Once inside the pig’s intestine, the eggs release oncospheres that hatch, penetrate the intestinal wall, and migrate to the striated muscles. There, they develop into larval cysts (cysticerci) over approximately 60–90 days. When infected pigs are slaughtered, cysticerci present in the muscle tissue result in contaminated pork entering the human food chain (Omeragić et al., 2023). If such pork is consumed raw or undercooked, viable cysticerci are ingested by humans, completing the transmission cycle.

*Taenia solium* Taeniasis is endemic in many regions of the world, including Asia, Latin America, and Sub-Saharan Africa, particularly in settings where pigs have access to human feces and pork consumption is common (Mwasunda, 2022). Several Researchers agree that it is a neglected health problem in impoverished communities in the endemic region with epidemiological studies estimating 2.56 million human Taeniasis cases worldwide, including at least 400,000 symptomatic cases in South America, and 1.5–3 million cases in sub-Saharan Africa (Omeragić et al., 2023).

The transmission of infectious diseases have long been of significant interest to both medical practitioners and researchers (Okello and Thomas, 2017) as further explained in Nyang’inja et al. (2018). Substantially, public health programmes play a great role in identifying and addressing health challenges, as they serve as primary sources of information and strongly influence behavioural responses within the population (Nyang’inja et al., 2018). Consequently, it is essential to study epidemic transmission dynamics and to develop effective strategies for prevention, control, and containment of *Taenia solium* Taeniasis. Individual responses to disease threats are largely shaped by risk perception, which is primarily informed by official reports disseminated by governmental and public health authorities. Such reports typically include data on the number of infections, hospitalizations, and fatalities provided by public health departments (Tchuenche et al., 2011, Nyang’inja et al., 2018). These epidemiological indicators play a critical role in shaping public behaviour and compliance with intervention measures. From a policy perspective, public health officials are particularly concerned with key epidemic metrics, such as the peak number of infections, the timing of the peak, the cumulative number of cases, and the duration or eventual end of the outbreak. These measures are closely associated with the planning, allocation, and management of essential healthcare resources (Mukandavire et al., 2009).

Over the years, mathematical models have served as a fundamental framework for analysing the transmission dynamics and for making necessary interventions regarding several infectious disease . According to Nyang’inja et al. (Nyang’inja et al., 2018), a general susceptible–infected–recovered or removed model studied in Huo and Wang (2016) was used to depict the transmission rate decreased by awareness intervention. Their findings demonstrated that effective awareness measures can reduce the number of infections, highlighting an effective method of reducing the spread of the disease being to make people understand the preventive measures as soon as possible. Mukandavire and Tchuenche (Mukandavire et al., 2009) formulated a mathematical model to study the effects of public health educational campaigns as a single control strategy on sexual transmission of Human Immunodeficiency Virus–Acquired Immunodeficiency Syndrome (HIV/AIDS) in the continuing absence of a preventative vaccine. The findings from this study underscore the significance of using public health educational campaigns in slowing down the infections in the population. The study in Haringo et al. (2024) introduced a compartmental mathematical model to investigate the impact of insecticide use and malaria treatment driven by awareness initiatives. Their framework incorporated the influence of media-based awareness on the effective utilization of insecticides for malaria control. The results showed that this integrated approach not only enhances the mathematical robustness of the model but also highlights its practical implications for developing effective malaria control strategies based on media-driven awareness.

Similar to Tungiasis modelling (see, e.g., Nyang’inja et al. (2018)), despite the significant harm caused by *Taenia solium* Taeniasis and the numerous challenges it poses to affected populations, studies addressing the disease from a mathematical modelling perspective are still limited. The several current studies so far conducted on *Taenia solium* Taeniasis have focused on the knowledge pertaining to the biology, epidemiology, and pathology of the *Taenia solium*, contributing factors of the disease, attitude and practices on the prevalence in different endemic regions (Okello and Thomas, 2017, Del Brutto and García, 2013, Zemedhun et al., 2025). However, only a limited number of studies in the literature have addressed this problem from a mathematical perspective. Recently, the authors in Mwasunda et al., 2021, Mwasunda, 2022 spearheaded the application of mathematical modelling to the transmission dynamics of Taeniasis. They formulated and analysed a compartmental model describing Taeniasis transmission in zoonotic settings and performed a sensitivity analysis to identify parameters with the greatest influence on the basic reproduction number, $R_{0}$, thereby highlighting potential targets for control interventions. Their findings indicate that reducing both the eggs and larvae concentration from the contaminated environment, as well as decreasing the effective contact rate between eggs/larvae sources and hosts, would constitute effective intervention strategies for mitigating the risk and magnitude of large transient Taeniasis consequences. (Carabin et al., 2016) extended the deterministic approach by developing a model to evaluate the impact of different intervention strategies on *Taenia solium* prevalence. Their work highlighted the importance of improving sanitation and pig husbandry practices to reduce transmission, leaving the environment factors which is reported to be the big accelerator of this disease. In literature (Winskill et al., 2017, Mwasunda, 2022), authors made a significant contribution to the literature by proposing the deterministic mathematical models to study the impacts of various strategies on the control of Taeniasis and Cysticercosis in humans and pigs. The pig population was divided into susceptible, vaccinated, pigs harbouring low cyst burdens, pigs harbouring high cyst burdens, and recovered pigs, while the human population was divided into susceptible, humans with Taeniasis, humans with Cysticercosis, and humans with both Taeniasis and Cysticercosis. Intervention strategies that were implemented singly or combined include pig vaccination, treatment of infected pigs, improved animal husbandry, improved meat inspection, and treatment of humans with Taeniasis. The results showed that treatment interventions that focus on the human or pig population are more effective in disease control when used alone, with annual treatment of pigs and humans. Collectively, these modelling efforts have provided valuable insights into the mechanisms underlying Taeniasis transmission dynamics, while also underscoring the need for improved empirical data to support the formulation, calibration, and validation of future models. Disease transmission has been extensively investigated in both classical compartmental modelling frameworks (José et al., 2018, Mwasunda et al., 2021, Rwabona et al., 2024), and other approaches, see for instance (Braae et al., 2016). To the best of our knowledge, no existing model has thoroughly examined the transmission dynamics of *Taenia solium* Taeniasis using mathematical modelling while explicitly incorporating public health education as an additional compartment for disease control. This gap in the literature constitutes the primary motivation for the present study. Following the framework presented in Nyang’inja et al. (2018), we theoretically investigate the dynamics of *Taenia solium* Taeniasis by incorporating public health education through a system of ordinary differential equations and computational simulations. In particular, we formulate the epidemic threshold to determine whether the infection will persist in the population, leading to an endemic equilibrium, or eventually die out, resulting in a disease-free equilibrium. This enables us to predict the potential disease burden in the community.

This work is organized as follows: Section 1 provides the background against the Taeniasis and the gap in the mathematical modelling of the disease. In Section 2, we formulate and describe the mathematical model. Section 3 presents the qualitative analysis results of the model. Section 4 presents numerical simulations to verify our theoretical results with their discussions. Finally, we conclude this work in Section 5.

## 2 Model formulation and assumptions

Following the compartmental framework established in Nyang’inja et al. (2018), we describe the transmission dynamics of Taenia solium within a community where humans, porcine hosts, and a contaminated environment coexist. The total human population, denoted by $N_{h}\left(t\right)$, is partitioned into four distinct health states: susceptible individuals ($S_{h}$), those who have received health education (A), exposed individuals in the latent stage ($E_{h}$), and infectious individuals ($I_{h}$).

Similarly, the porcine population $N_{p}\left(t\right)$ is divided into three compartments: susceptible ($S_{p}$), exposed ($E_{p}$), and infected ($I_{p}$). To represent the environmental reservoir of the parasite, we define two additional sub-compartments: E(t), representing the concentration of *T. solium* eggs (oncospheres) in the environment, and M(t), representing the density of cysticerci larvae in the food supply (infected pork).

A critical feature of this formulation is the inclusion of the exposed classes ($E_{h},E_{p}$). As noted by Winskill et al. (2017), a latent period of approximately 8–14 weeks is required post-exposure before a host becomes infectious; thus, the parameter θ is incorporated to capture the intrinsic biological delay in infectivity.

### 2.1 Population dynamics and transmission

The recruitment into the susceptible human and porcine populations occurs at constant rates, $\Lambda_{h}$ and $\Lambda_{p}$, respectively, through birth or immigration. Susceptible humans ($S_{h}$) transition to the exposed class ($E_{h}$) upon the ingestion of Taenia larvae (M) from the contaminated food environment at an effective transmission rate $\beta_{h}$. Conversely, susceptible pigs ($S_{p}$) enter the exposed class ($E_{p}$) through the ingestion of eggs (E) from the environment at rate $\beta_{p}$. Once the latent period concludes, exposed individuals progress to the infectious compartments ($I_{h}$ and $I_{p}$) at rates $\theta_{h}$ and $\theta_{p}$, respectively. We assume that humans do not acquire permanent immunity; thus, infectious individuals recover naturally at rate $\gamma_{h}$ and return directly to the susceptible class (Winskill et al., 2017). In contrast, infected pigs remain infectious for life unless treated or slaughtered, though they remain subject to natural mortality at rate $\mu_{p}$. All human compartments experience a natural mortality rate denoted by $\mu_{h}$.

### 2.2 Environmental contamination

The environmental reservoir is maintained by two primary pathways:

1. Egg Contamination (E): Infectious humans contribute to the environmental egg concentration through open defecation or poor hygiene at a rate $\sigma_{h}$. These eggs decay or are removed naturally at rate $\mu_{e}$.
2. Larval Contamination (M): The density of larvae in the food chain increases when infected pigs are slaughtered for consumption at rate $\sigma_{p}$. The infectiousness of the meat decreases or is removed (through inspection or spoilage) at rate $\mu_{m}$.

### 2.3 Modelling behavioural interventions and knowledge attrition

To evaluate the impact of non-pharmaceutical interventions (NPIs), we incorporate a transition from the susceptible class $S_{h}$ to the ‘Aware’ class A(t) at a rate ɛ. This parameter characterizes the effectiveness of public health education and the subsequent adoption of preventive behaviours. Recognizing that the influence of such education is often transient and that behavioural strategies may be only partially adopted, the model assumes that protective knowledge may diminish over time.

Consequently, individuals in the ‘Aware’ class (A) remain susceptible to infection, albeit at a significantly reduced rate. This attenuated force of infection is defined as $\alpha \beta_{h}AM$, where the scaling factor α (0≤α≤1) represents the residual risk following education. Specifically, α=0 denotes a scenario where education provides absolute protection (100% efficacy), whereas α=1 implies that the intervention provides no protective effect on transmission dynamics (Nyang’inja et al., 2018, Althubyani, 2021).

Furthermore, to account for the temporal decay of information retention — where individuals may revert to high-risk behaviours — we introduce the parameter ρ∈[0,1], representing the ’knowledge-decay’ rate (Mshana et al., 2023). This parameter facilitates the transition of individuals from the ‘Aware’ class (A) back to the susceptible human population ($S_{h}$). By incorporating both the efficacy coefficient α and the attrition rate ρ, the model distinguishes between the quality of the educational intervention and its durability over time. Consequently, the human population exists in a state of behavioural flux, where the aggregate infection risk is determined by the equilibrium between the uptake of preventive strategies (ɛ) and the natural attrition of adherence (ρ).

**Fig. 1:**
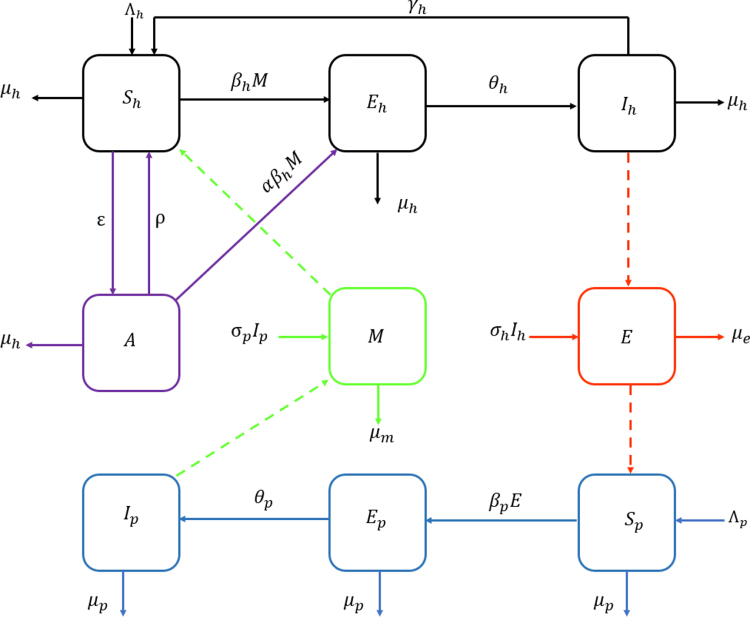
Schematic diagram illustrating the compartmental transitions and educational intervention pathways of the mathematical model.

The schematic representation of the model’s compartmental transitions is depicted in Fig. 1, while the baseline parameter values and their corresponding definitions are detailed in Table 1.

The compartmental transitions and behavioural pathways described in Section 2 are visually summarized in the schematic diagram presented in Fig. 1. Based on these biological and behavioural assumptions, the transmission dynamics of *T. solium* under a public health education framework are governed by the following system of non-linear ordinary differential equations:

(1) $$\left(\begin{array}{cc} \frac{dS_{h}}{dt} & =\Lambda_{h}-\left(\lambda_{h}+ɛ+\mu_{h}\right)S_{h}+\rho A+\gamma_{h}I_{h},\\ \frac{dA}{dt} & =ɛS_{h}-\left(\alpha \lambda_{h}+\rho +\mu_{h}\right)A,\\ \frac{dE_{h}}{dt} & =\lambda_{h}S_{h}+\alpha \lambda_{h}A-\left(\theta_{h}+\mu_{h}\right)E_{h},\\ \frac{dI_{h}}{dt} & =\theta_{h}E_{h}-\left(\gamma_{h}+\mu_{h}\right)I_{h},\\ \frac{dS_{p}}{dt} & =\Lambda_{p}-\left(\lambda_{p}+\mu_{p}\right)S_{p},\\ \frac{dE_{p}}{dt} & =\lambda_{p}S_{p}-\left(\theta_{p}+\mu_{p}\right)E_{p},\\ \frac{dI_{p}}{dt} & =\theta_{p}E_{p}-D_{5}I_{p}.\\ \frac{dM}{dt} & =\sigma_{p}I_{p}-\mu_{m}M,\\ \frac{dE}{dt} & =\sigma_{h}I_{h}-\mu_{e}E, \end{array}\right\}$$

where: $\lambda_{h}=\beta_{h}M$, $\lambda_{p}=\beta_{p}E$. To facilitate the subsequent stability analysis and streamline the algebraic complexity, we introduce the following effective removal rates:

$$D_{1}=\epsilon +\mu_{h},\;D_{2}=\theta_{h}+\mu_{h},\;D_{3}=\gamma_{h}+\mu_{h},\;D_{4}=\theta_{p}+\mu_{p},\;D_{5}=\alpha_{p}+\mu_{p}.$$

Under these notations, the system of differential Eqs. (1) is expressed in the following compact form:

(2) $$\left(\begin{array}{cc} \frac{dS_{h}}{dt} & =\Lambda_{h}+\gamma_{h}I_{h}-\left(\lambda_{h}+D_{1}\right)S_{h}+\rho A,\\ \frac{dA}{dt} & =ɛS_{h}-\alpha \lambda_{h}A-\left(\rho +\mu_{h}\right)A,\\ \frac{dE_{h}}{dt} & =\lambda_{h}S_{h}+\alpha \lambda_{h}A-D_{2}E_{h},\\ \frac{dI_{h}}{dt} & =\theta_{h}E_{h}-D_{3}I_{h},\\ \frac{dS_{p}}{dt} & =\Lambda_{p}-\lambda_{p}S_{p}-\mu_{p}S_{p},\\ \frac{dE_{p}}{dt} & =\lambda_{p}S_{p}-D_{4}E_{p},\\ \frac{dI_{p}}{dt} & =\theta_{p}E_{p}-D_{5}I_{p},\\ \frac{dM}{dt} & =\sigma_{p}I_{p}-\mu_{m}M,\\ \frac{dE}{dt} & =\sigma_{h}I_{h}-\mu_{e}E. \end{array}\right\}$$

Given that system (2) describes the evolution of human and porcine populations, it is imperative that all state variables and their associated parameters remain non-negative for all t≥0 to maintain biological feasibility. In the following subsection, we establish the mathematical well-posedness of the model by proving the non-negativity and boundedness of its solutions within a defined positively invariant region. To further elucidate the mechanisms driving the transmission of *T. solium* and to characterize the long-term asymptotic behaviour of the disease, we conduct a qualitative analysis of the proposed system. This analysis provides the theoretical foundation for determining the stability of the equilibrium points and identifying the threshold conditions under which the infection either persists or is successfully eliminated from the population.

**Table 1:** Model parameters: Definitions, baseline values, and sources (Units: day^−1^ unless otherwise specified).

Symbol	Description	Value	Source
$\Lambda_{h}$	Recruitment rate of humans	187	Mwasunda et al. (2021)
$\Lambda_{p}$	Recruitment rate of pigs	121	Mwasunda et al. (2021)
ɛ	Rate of public health intervention and education	$1.431\times 10^{-3}$	Nyang’inja et al. (2018)
α	Efficacy of education in reducing infection risk	0 – 1	Nyang’inja et al. (2018)
ρ	Rate of knowledge decay (loss of adherence)	0.00027	Mshana et al. (2023)
$\mu_{h}$	Natural death rate of humans	0.0141	Rwabona et al. (2024)
$\gamma_{h}$	The natural clearance rate of *T. solium* adult worms in the human host.	0.001369	Kywsgaard et al., 2007, Centers for Disease Control and Prevention, 2024
$\mu_{p}$	Natural death rate of pigs	0.083	Rwabona et al. (2024)
$\mu_{e}$	Environmental clearance rate of *T. solium* eggs	0.0667	Rwabona et al. (2024)
$\mu_{m}$	Environmental clearance rate of *T. solium* larvae	0.0812	Rwabona et al. (2024)
$\theta_{h}$	Progression rate from exposed to infectious humans	0.0119	Willis and Herbert (2014)
$\theta_{p}$	Progression rate from exposed to infectious pigs	$7.143\times 10^{-3}$	Willis and Herbert (2014)
$\beta_{h}$	Transmission rate from environment to humans	$1.4\times 10^{-4}$	Rwabona et al. (2024)
$\beta_{p}$	Transmission rate from environment to pigs	$7.86\times 10^{-7}$	Rwabona et al. (2024)
$\sigma_{h}$	Environmental egg contamination rate	$3.2\times 10^{5}$	Irunde and Luhanda (2023)
$\sigma_{p}$	Environmental larval contamination rate	1000 cysts/kg	Kabululu et al. (2022)

## 3 Analysis of model

To gain insights into the underlying mechanisms that drive the spread of infectious *Taenia solium* Taeniasis to identify the long-term behaviour of disease transmission we conduct qualitative analysis of the proposed model (2).

### 3.1 Positivity and boundedness of solutions

To ensure the biological relevance of the model (2), it is necessary to prove that all state variables remain non-negative and bounded for all t≥0. Following the mathematical framework established by Althubyani (2021), we define the biologically feasible region Ω and establish the following theorem regarding the existence and confinement of solutions:

Theorem 1 *Let the initials conditions of model* (2) *be*

$$S_{h}\left(0\right)>0,A\left(0\right)>0,E_{h}\left(0\right)\geq 0,I_{h}\left(0\right)\geq 0,$$

$$S_{p}\left(0\right)>0,E_{p}\left(0\right)\geq 0,I_{p}\left(0\right)\geq 0,M\left(0\right)\geq 0,E\left(0\right)\geq 0.$$

*The solutions*

$$S_{h}\left(t\right),A\left(t\right),E_{h}\left(t\right),I_{h}\left(t\right),S_{p}\left(t\right),E_{p}\left(t\right),I_{p}\left(t\right),M\left(t\right),E\left(t\right)$$

*of model* (2)*, with positive initial conditions, remain positive for all* t>0*.*

Proof Let

$$t_{1}=\sup\left\{t>0:S_{h}>0,A>0,E_{h}\geq 0,I_{h}\geq 0,S_{p}>0,E_{p}\geq 0,I_{p}\geq 0,M\geq 0,E\geq 0\right\}>0.$$

That is, since the involved populations are living things, all the sub populations must be non-negative and the positivity must not fail in order to for the model to be biologically meaningful.

From the first equation of system (2), is

$$\Lambda_{h}+\gamma_{h}I_{h}+\rho A-\left(\lambda_{h}-D_{1}\right)S_{h},$$

which can be written as

(3) $$\gamma_{h}I_{h}+\rho A+\lambda_{h}S_{h}+D_{1}S_{h}\leq \Lambda_{h}.$$

Solving Eq. (3) by integrating factor, gives

(4) $$\psi \leq \exp\left(D_{1}t+\int_{0}^{t}\lambda_{h}\left(\tau \right)d\tau \right).$$

Multiplying through by ψ, and after some simplification, yields

(5) $$\frac{d}{dt}\left[S_{h}\left(t\right)\exp\left(D_{1}t+\int_{0}^{t}\lambda_{h}\left(\tau \right)d\tau \right)\right]\leq \Lambda_{h}\left[\exp\left(D_{1}t+\int_{0}^{t}\lambda_{h}\left(\tau \right)d\tau \right)\right].$$

Thus, by integrating from t=0 to $t=t_{1}$ results into

(6) $$S_{h}\left(t_{1}\right)\geq S_{h}\left(0\right)\exp\left(-D_{1}t_{1}-\int_{0}^{t_{1}}\lambda_{h}\left(\tau \right)d\tau \right)\;\;\;\;+\Lambda_{h}\exp\left(-D_{1}t_{1}-\int_{0}^{t_{1}}\lambda_{h}\left(\tau \right)d\tau \right)\int_{0}^{t_{1}}\exp\left(D_{1}s+\int_{0}^{s}\lambda_{h}\left(\tau \right)d\tau \right)ds>0.$$

In similar manner, the remaining equations of model (2) are expressed as:

Using the differential inequality method, we observe that for the remaining each state variable, the rate of change at the boundary of the non-negative orthant satisfies:

$$\frac{dA}{dt}\geq -\mu_{h}A⟹A\left(t\right)>0,$$

$$\frac{dE_{h}}{dt}\geq -D_{2}E_{h}⟹E_{h}\left(t\right)\geq 0,$$

$$\frac{dI_{h}}{dt}\geq -D_{3}I_{h}⟹I_{h}\left(t\right)\geq 0,$$

$$\frac{dS_{p}}{dt}\geq -\mu_{p}S_{p}⟹S_{p}\left(t\right)>0,$$

$$\frac{dE_{p}}{dt}\geq -D_{4}E_{p}⟹E_{p}\left(t\right)\geq 0,$$

$$\frac{dI_{p}}{dt}\geq -D_{5}I_{p}⟹I_{p}\left(t\right)\geq 0,$$

$$\frac{dM}{dt}\geq -\mu_{m}A⟹M\left(t\right)\geq 0,$$

$$\frac{dE}{dt}\geq -\mu_{m}A⟹E\left(t\right)\geq 0,$$

provided that the initial conditions $A\left(0\right)>0,E_{h}\left(0\right)\geq 0,I_{h}\left(0\right)\geq 0,S_{p}\left(0\right)>0,E_{p}\left(0\right)\geq 0,I_{p}\left(0\right)\geq 0,M\left(0\right)\geq 0,E\left(0\right)\geq 0$.

To ensure the biological and mathematical consistency of the system, the dynamics of model (2) are analysed within a compact, positively invariant feasible region. This region, denoted by D, is defined as follows:

(7) $$D=\left\{\chi \in R_{+}^{9}:\begin{array}{c} N_{h}\left(t\right)\leq \frac{\Lambda_{h}}{\mu_{h}},\\ N_{p}\left(t\right)\leq \frac{\Lambda_{p}}{\mu_{p}},\\ M\left(t\right)\leq \frac{\sigma_{p}\Lambda_{p}}{\mu_{m}\mu_{p}},\\ E\left(t\right)\leq \frac{\sigma_{h}\Lambda_{h}}{\mu_{e}\mu_{h}} \end{array}\right\},$$

where

$$\chi =\left(S_{h},A,E_{h},I_{h},S_{p},E_{p},I_{p},M,E\right),$$

with $N_{h}\left(t\right)=S_{h}\left(t\right)+A\left(t\right)+E_{h}\left(t\right)+I_{h}\left(t\right)$ and $N_{p}\left(t\right)=S_{p}\left(t\right)+E_{p}\left(t\right)+I_{p}\left(t\right)$.

Lemma 2 *The domain* D *is positively invariant and a global attractor for all positive solutions of the system* (2)*.*

Proof Following the qualitative analysis framework for epidemic models described in Althubyani (2021), we first establish the boundedness of the system’s solutions. By summing the first four equations of the model system (2), we obtain the dynamics of the total human population size as follows:

(8) $$N_{h}\left(t\right)=S_{h}\left(t\right)+A\left(t\right)+E_{h}\left(t\right)+I_{h}\left(t\right).$$

Yields,

$$\frac{dN_{h}}{dt}=\frac{dS_{h}}{dt}+\frac{dA}{dt}+\frac{dE_{h}}{dt}+\frac{dI_{h}}{dt}$$

(9) $$\frac{dN_{h}}{dt}\leq \Lambda_{h}-\mu_{h}N_{h}.$$

Similarly, pig population gives

(10) $$\frac{dN_{p}}{dt}\leq \Lambda_{p}-\mu_{h}N_{p}.$$

By considering the total population of humans, pigs and cattle separately, it follows that

(11) $$\left\{\begin{array}{c} \frac{dN_{h}}{dt}\leq \Lambda_{h}-\mu_{h}N_{h}\\ \frac{dN_{p}}{dt}\leq \Lambda_{p}-\mu_{h}N_{p} \end{array}\;\;\right)$$

From Eq. (11), using integration technique, the first inequality leads to

(12) $$N_{h}\left(t\right)\leq \frac{\Lambda_{h}}{\mu_{h}}-\left(N_{h}\left(0\right)-\frac{\Lambda_{h}}{\mu_{h}}\right)e^{-\mu_{h}t}.$$

Applying the standard comparison theorem (Lakshmikantham et al., 1989), when $N_{h}\left(0\right)>\frac{\Lambda_{h}}{\mu_{h}}$ and $N_{h}\left(0\right)<\frac{\Lambda_{h}}{\mu_{h}}$, yields

(13) $$\left\{\begin{array}{c} \frac{\Lambda_{h}}{\mu_{h}}\leq N_{h}\left(t\right)\leq \frac{\Lambda_{h}}{\mu_{h}}+\left(N_{h}\left(0\right)-\frac{\Lambda_{h}}{\mu_{h}}\right)e^{-\mu_{h}t},\\ \frac{\Lambda_{h}}{\mu_{h}}+\left(N_{h}\left(0\right)-\frac{\Lambda_{h}}{\mu_{h}}\right)e^{-\mu_{h}t}\leq N_{h}\left(t\right)\leq \frac{\Lambda_{h}}{\mu_{h}}. \end{array}\;\;\right)$$

Similarly, following the same argument, other inequalities in Eq. (11), give

(14) $$\left\{\begin{array}{c} \frac{\Lambda_{p}}{\mu_{p}}\leq N_{p}\left(t\right)\leq \frac{\Lambda_{p}}{\mu_{p}}+\left(N_{p}\left(0\right)-\frac{\Lambda_{p}}{\mu_{p}}\right)e^{-\mu_{p}t},\\ \frac{\Lambda_{p}}{\mu_{p}}+\left(N_{p}\left(0\right)-\frac{\Lambda_{p}}{\mu_{p}}\right)e^{-\mu_{p}t}\leq N_{p}\left(t\right)\leq \frac{\Lambda_{p}}{\mu_{p}}, \end{array}\right)$$

When time t approaching to infinity, it follows that

(15) $$\left\{\begin{array}{c} 0\leq N_{h}\left(t\right)\leq \frac{\Lambda_{h}}{\mu_{h}},\\ 0\leq N_{p}\left(t\right)\leq \frac{\Lambda_{p}}{\mu_{p}}, \end{array}\right)$$

Finally, we consider the environmental egg concentration E and the infected meat concentration M. Since $I_{h}$ and $I_{p}$ are bounded by $\frac{\Lambda_{h}}{\mu_{h}}$ and $\frac{\Lambda_{p}}{\mu_{p}}$ respectively, we have:

(16) $$\frac{dE}{dt}=\sigma_{h}I_{h}-\mu_{e}E\leq \sigma_{h}\left(\frac{\Lambda_{h}}{\mu_{h}}\right)-\mu_{e}E,$$

(17) $$\frac{dM}{dt}=\sigma_{p}I_{p}-\mu_{m}M\leq \sigma_{p}\left(\frac{\Lambda_{p}}{\mu_{p}}\right)-\mu_{m}M.$$

Applying the same differential inequality logic, it follows that:

(18) $$E\left(t\right)\leq \frac{\sigma_{h}\Lambda_{h}}{\mu_{e}\mu_{h}}\;\text{and}\;M\left(t\right)\leq \frac{\sigma_{p}\Lambda_{p}}{\mu_{m}\mu_{p}}.$$

Thus, $D_{1}$ attracts all the solutions in $R^{9}$. Since the domain $D_{1}$ is positively-invariant, it is sufficient to consider the dynamics of model (2) in $D_{1}$. In this region, the model (2) can be considered as being both epidemiologically and mathematically well posed (Althubyani, 2021).

### 3.2 Disease-free equilibrium (DFE)

The system (2) possesses two distinct disease-free equilibrium points, corresponding to the absence and presence of public health interventions. The DFE represents a steady state where the infection is eliminated from both the human and porcine populations. This state is obtained by setting all infectious and latent compartments to zero, such that $E_{h}=I_{h}=E_{p}=I_{p}=E=M=0$, and solving the remaining algebraic equations for the steady state.

### 3.3 Case I: DFE in the absence of education (ɛ=0,ρ=0)

In a scenario where no public health education is disseminated, the population remains entirely susceptible. The equilibrium point, denoted as $E_{0}$, is given by:

(19) $$E_{0}=\left(\frac{\Lambda_{h}}{\mu_{h}},0,0,0,\frac{\Lambda_{p}}{\mu_{p}},0,0,0,0\right).$$

### 3.4 Case II: DFE with education (ɛ>0)

When public health interventions are active, the susceptible human population is partitioned between the uninformed ($S_{h}$) and the aware (A) classes. Setting the derivatives in (2) to zero under disease-free conditions yields the following system:

(20) $$\Lambda_{h}+\rho A^{0}-\left(ɛ+\mu_{h}\right)S_{h}^{0}=0$$

(21) $$ɛS_{h}^{0}-\left(\rho +\mu_{h}\right)A^{0}=0$$

Solving this system simultaneously, we obtain the coordinates for the stable aware population:

(22) $$S_{h}^{0}=\frac{\Lambda_{h}\left(\rho +\mu_{h}\right)}{\mu_{h}\left(ɛ+\rho +\mu_{h}\right)},\;A^{0}=\frac{ɛ\Lambda_{h}}{\mu_{h}\left(ɛ+\rho +\mu_{h}\right)}$$

Thus, the disease-free equilibrium point under the influence of education, $E_{ɛ}$, is defined as:

(23) $$E_{ɛ}=\left(S_{h}^{0},A^{0},0,0,\frac{\Lambda_{p}}{\mu_{p}},0,0,0,0\right).$$

### 3.5 Threshold analysis: The effective reproduction number

The potential for disease transmission and persistence within the population is characterized by two fundamental epidemiological quantities: the basic reproduction number, $R_{0}^{\left(L\right)}$, and the education-influenced effective reproduction number, $R_{ed}$. These metrics represent the average number of secondary infections generated by a single infectious individual introduced into a susceptible population in the absence and presence of control measures, respectively (Rwabona et al., 2024, Althubyani, 2021).

To derive these thresholds for system (2), we employ the Next-Generation Matrix (NGM) method as described by Van den Driessche and Watmough (2002). We consider the vector of infected classes $X={\left[E_{h},I_{h},E_{p},I_{p},E,M\right]}^{T}$. The transmission dynamics are decomposed into the rate of new infections, F, and the rate of transfer between compartments, V, evaluated at the disease-free equilibria $E_{0}$ and $E_{ɛ}$ respectively.

By linearizing the system (2) at the DFE, the transmission matrix F and transition matrix V are given by:

(24) $$F=\left(\begin{array}{cccccc} 0 & 0 & 0 & 0 & \alpha \beta_{h}A_{0}+\beta_{h}S_{h0} & 0\\ 0 & 0 & 0 & 0 & 0 & 0\\ 0 & 0 & 0 & 0 & 0 & \beta_{p}S_{p0}\\ 0 & 0 & 0 & 0 & 0 & 0\\ 0 & 0 & 0 & 0 & 0 & 0\\ 0 & 0 & 0 & 0 & 0 & 0 \end{array}\right),V=\left(\begin{array}{cccccc} D_{2} & 0 & 0 & 0 & 0 & 0\\ -\theta_{h} & D_{3} & 0 & 0 & 0 & 0\\ 0 & 0 & D_{4} & 0 & 0 & 0\\ 0 & 0 & -\theta_{p} & D_{5} & 0 & 0\\ 0 & 0 & 0 & -\sigma_{p} & \mu_{m} & 0\\ 0 & -\sigma_{h} & 0 & 0 & 0 & \mu_{e} \end{array}\right).$$

Thus,

(25) $$V^{-1}=\left(\begin{array}{cccccc} \frac{1}{D_{2}} & 0 & 0 & 0 & 0 & 0\\ \frac{\theta_{h}}{D_{2}D_{3}} & \frac{1}{D_{3}} & 0 & 0 & 0 & 0\\ 0 & 0 & \frac{1}{D_{4}} & 0 & 0 & 0\\ 0 & 0 & \frac{\theta_{p}}{D_{4}D_{5}} & \frac{1}{D_{5}} & 0 & 0\\ 0 & 0 & \frac{\sigma_{p}\theta_{p}}{D_{4}D_{5}\mu_{m}} & \frac{\sigma_{p}}{D_{5}\mu_{m}} & \frac{1}{\mu_{m}} & 0\\ \frac{\sigma_{h}\theta_{h}}{D_{2}D_{3}\mu_{e}} & \frac{\sigma_{h}}{D_{3}\mu_{e}} & 0 & 0 & 0 & \frac{1}{\mu_{e}} \end{array}\right).$$

The next generation Matrix $K=FV^{-1}$ is computed by mapping the transmission rates to the expected duration of stay in each infectious compartment:

(26) $$K=\left(\begin{array}{cccccc} K_{11} & K_{12} & 0 & 0 & 0 & K_{16}\\ 0 & 0 & 0 & 0 & 0 & 0\\ K_{31} & K_{32} & 0 & 0 & 0 & K_{36}\\ 0 & 0 & 0 & 0 & 0 & 0\\ 0 & 0 & 0 & 0 & 0 & 0\\ 0 & 0 & 0 & 0 & 0 & 0 \end{array}\right)$$

Where the non-zero entries are:

$$K_{11}=\frac{\left(\alpha \beta_{h}A_{0}+\beta_{h}S_{h0}\right)\sigma_{p}\theta_{p}\theta_{h}}{D_{2}D_{3}D_{4}D_{5}\mu_{m}},\;K_{12}=\frac{\left(\alpha \beta_{h}A_{0}+\beta_{h}S_{h0}\right)\sigma_{p}\theta_{p}}{D_{3}D_{4}D_{5}\mu_{m}},$$

$$K_{16}=\frac{\alpha \beta_{h}A_{0}+\beta_{h}S_{h0}}{\mu_{m}},\;K_{31}=\frac{\beta_{p}S_{p0}\sigma_{h}\theta_{h}}{D_{2}D_{3}\mu_{e}},$$

$$K_{32}=\frac{\beta_{p}S_{p0}\sigma_{h}}{D_{3}\mu_{e}},\;K_{36}=\frac{\beta_{p}S_{p0}}{\mu_{e}}.$$

Hence, the basic reproduction $R_{0}^{\left(L\right)}$ corresponds to $E_{0}$, in the absence of education intervention or public health intervention is

(27) $$R_{0}^{\left(L\right)}=\sqrt{\frac{\Lambda_{p}\Lambda_{h}\beta_{h}\beta_{p}\sigma_{h}\sigma_{p}\theta_{h}\theta_{p}}{\left(\theta_{h}+\mu_{h}\right)\left(\gamma_{h}+\mu_{h}\right)\left(\theta_{p}+\mu_{p}\right)\left(\alpha_{p}+\mu_{p}\right)\mu_{h}\mu_{p}\mu_{m}\mu_{e}}}.$$

While the education-dependent reproduction $R_{ed}$ for DFE, $E_{ɛ}$ in the presence of public health intervention is defined as:

(28) $$R_{ed}=\sqrt{\left(\frac{\left(\rho +\mu_{h}\right)+\alpha ɛ}{ɛ+\rho +\mu_{h}}\right)\frac{\Lambda_{p}\Lambda_{h}\beta_{h}\beta_{p}\sigma_{h}\sigma_{p}\theta_{h}\theta_{p}}{\left(\theta_{h}+\mu_{h}\right)\left(\gamma_{h}+\mu_{h}\right)\left(\theta_{p}+\mu_{p}\right)\left(\alpha_{p}+\mu_{p}\right)\mu_{h}\mu_{p}\mu_{m}\mu_{e}}}.$$

Where Φ is the education impact factor:

$$\Phi \left(ɛ,\rho ,\alpha \right)=\frac{\left(\rho +\mu_{h}\right)+\alpha ɛ}{ɛ+\rho +\mu_{h}}.$$

Then:

(29) $$R_{ed}=\sqrt{\Phi }R_{0}^{\left(L\right)}.$$

Given that the parameter 0<ɛ<1 (since α<1), it follows that the reduction factor Φ is strictly less than unity (Φ<1). This inequality implies that the reproduction number under the education model, $R_{ed}$, is consistently lower than the baseline reproduction number, $R_{0}^{\left(L\right)}$. As demonstrated in Eq. (29), these results rigorously show that public health education interventions exert a positive impact by significantly reducing the rate of new *T. solium* infections within the population.

We further elucidate the mechanisms by which the education parameters govern the transmission dynamics of *T. solium* through the reduction factor Φ:

• **Case of No Education** (ɛ=0): In the absence of a public health campaign, the intervention parameter ɛ vanishes, leading to Φ=1. Consequently, the control reproduction number $R_{ed}$ reverts to the baseline basic reproduction number $R_{0}^{\left(L\right)}$, representing a scenario with no behavioural modification.
• **Idealized Education** (α=0 and ρ=0): Under the assumption of perfect efficacy (where educated individuals are entirely immune to infection, α=0) and permanent retention of knowledge (no behavioural decay, ρ=0), the threshold is reduced proportionally to the ratio of susceptible individuals who remain in the $S_{h}$ class versus those who successfully transition to the “Aware” class A.
• **Optimization of the Threshold:** The analytical structure of Φ demonstrates that to minimize the epidemic threshold, the public health strategy must focus on maximizing the recruitment rate into the educated class (ɛ) while simultaneously minimizing the rate of behavioural decay or “forgetting” (ρ).
• **Impact of Imperfect Awareness** (α>0): Even in scenarios where education is imperfect — meaning aware individuals still face a residual risk of infection — the “Aware” compartment A serves as a critical epidemiological buffer. By reducing the effective force of infection, this buffer ensures that $R_{ed}$ remains strictly lower than $R_{0}^{\left(L\right)}$, thereby slowing the overall spread of the disease.

Consequently, the threshold quantities $R_{0}^{\left(L\right)}$ and $R_{ed}$ serve as the reproductive metrics for the dynamical system (2). Specifically, $R_{0}^{\left(L\right)}$ denotes the basic reproduction number, representing the expected number of secondary infections generated by a single infectious individual in a completely susceptible population in the absence of public health education or behavioural interventions. In contrast, $R_{ed}$ represents the control reproduction number, which quantifies the transmission potential when public health education and awareness campaigns are actively implemented. Comparing these two thresholds allows for a quantitative assessment of the intervention’s efficacy in curtailing the spread of *T. solium*. This determination agreed with the published results done by Althbyani (Althubyani, 2021).

### 3.6 Local stability DFE

Theorem 3 *The Disease-Free Equilibrium (DFE) of the system* (2) *is locally asymptotically stable if* $R_{ed}<1$*, and unstable if* $R_{ed}>1$*.*

Proof The local asymptotic stability of the disease-free equilibrium (DFE), denoted by $E_{ɛ}$, is fundamentally determined by the spectral properties of the Jacobian matrix evaluated at that equilibrium (Rwabona et al., 2024). In accordance with the next-generation matrix method, the linearized dynamics of the infected compartments — consisting of $E_{h},I_{h},E_{p},I_{p},M$, and E — are governed by the transmission matrix F and the transition matrix V, as explicitly defined in (24). By analysing the eigenvalues of the linearized system, we can establish the threshold conditions under which the pathogen is cleared from the population.

Following the criteria established in Van den Driessche and Watmough (2002), the DFE is locally asymptotically stable if all eigenvalues of the linearized system matrix J=F−V possess negative real parts. This condition is mathematically equivalent to the requirement that the spectral radius of the next-generation matrix, $\rho \left(FV^{-1}\right)$, is strictly less than unity. Consequently, the DFE is stable if $R_{ed}<1$, implying that any small-scale introduction of the pathogen into the population will result in its eventual extinction.

To show this explicitly, consider the characteristic equation det(F−V−λI)=0. Considering model system (2), the Jacobian matrix at the DFE takes the block form:

(30) $$J_{DFE}=\left(\begin{array}{cc} A_{11} & A_{12}\\ 0 & J_{infected} \end{array}\right)$$

where $A_{11}$ corresponds to the non-infected classes ($S_{h},A,S_{p}$). The eigenvalues of $A_{11}$ are $-\mu_{h},-\left(ɛ+\rho +\mu_{h}\right)$, and $-\mu_{p}$, all of which are strictly negative.

The remaining eigenvalues are determined by the sub-matrix, $J_{s}$ of $J_{infected}=F-V$. Since V in is a non-singular Metzeler matrix and F is non-negative, the stability of F−V is entirely governed by the threshold $R_{ed}$. Specifically, if $R_{ed}<1$, then $J_{s}\left(F-V\right)<0$ (where $J_{s}$ is the maximum real part of the eigenvalues).

Thus, all eigenvalues of the Jacobian matrix at the DFE have negative real parts if and only if $R_{ed}<1$. We conclude that the DFE is locally asymptotically stable when $R_{ed}<1$.

By applying the same analytical procedure used in Theorem 3, we establish the stability criteria for the baseline scenario. Specifically, in the absence of public health education (ɛ=0), the model (2) reduces to a baseline system where the following threshold dynamics apply:

Theorem 4 *The baseline Disease-Free Equilibrium,* $E_{0}$*, of the system* (2) *is locally asymptotically stable (LAS) if* $R_{0}^{\left(L\right)}<1$ *and unstable if* $R_{0}^{\left(L\right)}>1$*.*

### 3.7 Global stability of the Disease-Free Equilibrium (DFE)

To establish the global asymptotic stability of the disease-free equilibrium $E_{ɛ}$, we apply the framework developed by Castillo-(Castillo-Chavez et al., 2002). We partition the state variables of system (2) into uninfected compartments $X={\left(S_{h},A,S_{p}\right)}^{T}\in R^{3}$ and infected compartments $Y={\left(E_{h},I_{h},E_{p},I_{p},M,E\right)}^{T}\in R^{6}$.

The system is written as:

(31) $$\frac{dX}{dt}=F\left(X,Y\right),$$

$$\frac{dY}{dt}=G\left(X,Y\right),\;G\left(X,0\right)=0,$$

where $E_{ɛ}=\left(X^{*},0\right)$ is the DFE, with:

$$S_{h}^{*}=\frac{\Lambda_{h}\left(\rho +\mu_{h}\right)}{\mu_{h}\left(ɛ+\rho +\mu_{h}\right)},\;A^{*}=\frac{ɛ\Lambda_{h}}{\mu_{h}\left(ɛ+\rho +\mu_{h}\right)},\;S_{p}^{*}=\frac{\Lambda_{p}}{\mu_{p}}.$$

According to Castillo-(Castillo-Chavez et al., 2002), $E_{ɛ}$ is globally asymptotically stable if $R_{ed}<1$ provided the following two conditions hold:

**(H1)** For $\frac{dX}{dt}=F\left(X,0\right)$, $X^{*}$ is globally asymptotically stable.
**(H2)** $G\left(X,Y\right)=BY-\overset{ˆ}{G}\left(X,Y\right)$, where $B=D_{Y}G\left(X^{*},0\right)$ is an M-matrix and $\overset{ˆ}{G}\left(X,Y\right)\geq 0$ for all (X,Y)∈D.

Theorem 5 *The disease-free equilibrium* $E_{ɛ}$ *of model system* (2) *is globally asymptotically stable whenever* $R_{ed}<1$ *and unstable if* $R_{ed}>1$*.*

Proof To verify condition **(H1)**, we set Y=0, reducing the uninfected sub-system to:

$$\frac{dS_{h}}{dt}=\Lambda_{h}-D_{1}S_{h}+\rho A,$$

$$\frac{dA}{dt}=ɛS_{h}-\left(\rho +\mu_{h}\right)A,$$

$$\frac{dS_{p}}{dt}=\Lambda_{p}-\mu_{p}S_{p}.$$

This is a stable linear system operating within a closed, bounded region. As t→∞, the solutions carry a unique, global convergence directly to $X^{*}={\left(S_{h}^{*},A^{*},S_{p}^{*}\right)}^{T}$. Thus, condition **(H1)** is satisfied.

To verify condition **(H2)**, we compute the Jacobian matrix B of the infected sub-system evaluated at the DFE. Differentiating G(X,Y) with respect to Y at $\left(X^{*},0\right)$ yields:

$$A=\left(\begin{array}{cccccc} -D_{2} & 0 & 0 & 0 & \beta_{h}\left(S_{h}^{*}+\alpha A^{*}\right) & 0\\ \theta_{h} & -D_{3} & 0 & 0 & 0 & 0\\ 0 & 0 & -D_{4} & 0 & 0 & \beta_{p}S_{p}^{*}\\ 0 & 0 & \theta_{p} & -D_{5} & 0 & 0\\ 0 & 0 & 0 & \sigma_{p} & -\mu_{m} & 0\\ 0 & \sigma_{h} & 0 & 0 & 0 & -\mu_{e} \end{array}\right)$$

Using the Next-Generation Matrix approach, we decompose B into B=F−V, where F handles newly appearing infections and V handles compartmental transfers:

$$F=\left(\begin{array}{cccccc} 0 & 0 & 0 & 0 & \beta_{h}\left(S_{h}^{*}+\alpha A^{*}\right) & 0\\ 0 & 0 & 0 & 0 & 0 & 0\\ 0 & 0 & 0 & 0 & 0 & \beta_{p}S_{p}^{*}\\ 0 & 0 & 0 & 0 & 0 & 0\\ 0 & 0 & 0 & 0 & 0 & 0\\ 0 & 0 & 0 & 0 & 0 & 0 \end{array}\right),\;V=\left(\begin{array}{cccccc} D_{2} & 0 & 0 & 0 & 0 & 0\\ -\theta_{h} & D_{3} & 0 & 0 & 0 & 0\\ 0 & 0 & D_{4} & 0 & 0 & 0\\ 0 & 0 & -\theta_{p} & D_{5} & 0 & 0\\ 0 & 0 & 0 & -\sigma_{p} & \mu_{m} & 0\\ 0 & -\sigma_{h} & 0 & 0 & 0 & \mu_{e} \end{array}\right)$$

The matrix B is a stable M-matrix if and only if the spectral radius $\rho \left(FV^{-1}\right)<1$. Calculating the regular matrix product explicitly gives the generation loop value:

$$\rho \left(FV^{-1}\right)=\frac{\beta_{h}\beta_{p}\theta_{h}\theta_{p}\sigma_{h}\sigma_{p}\left(S_{h}^{*}+\alpha A^{*}\right)S_{p}^{*}}{D_{2}D_{3}D_{4}D_{5}\mu_{m}\mu_{e}}$$

Substituting $S_{p}^{*}=\frac{\Lambda_{p}}{\mu_{p}}$ and $S_{h}^{*}+\alpha A^{*}=\frac{\Lambda_{h}}{\mu_{h}}\Phi \left(ɛ,\rho ,\alpha \right)$, we find:

$$\rho \left(FV^{-1}\right)=\Phi \left(ɛ,\rho ,\alpha \right)\cdot \frac{\Lambda_{h}\Lambda_{p}\beta_{h}\beta_{p}\sigma_{h}\sigma_{p}\theta_{h}\theta_{p}}{D_{2}D_{3}D_{4}D_{5}\mu_{h}\mu_{p}\mu_{m}\mu_{e}}=R_{ed}^{2}$$

Thus, $\rho \left(FV^{-1}\right)<1$ is satisfied precisely when $R_{ed}<1$, ensuring all eigenvalues of B possess negative real parts.

Finally, we evaluate the remainder vector $\overset{ˆ}{G}\left(X,Y\right)=BY-G\left(X,Y\right)$, which yields:

$$\overset{ˆ}{G}\left(X,Y\right)=\left(\begin{array}{c} \beta_{h}M\left[\left(S_{h}^{*}-S_{h}\right)+\alpha \left(A^{*}-A\right)\right]\\ 0\\ \beta_{p}E\left(S_{p}^{*}-S_{p}\right)\\ 0\\ 0\\ 0 \end{array}\right)$$

Since the total human and pig populations are bounded by their natural carrying capacities inside the biologically feasible region D, it follows that $S_{h}\leq S_{h}^{*}$, $A\leq A^{*}$, and $S_{p}\leq S_{p}^{*}$. Therefore, $\overset{ˆ}{G}\left(X,Y\right)\geq 0$ holds true universally for all states in D.

Both conditions **(H1)** and **(H2)** are satisfied. By the Castillo-Chavez theorem, the disease-free equilibrium $E_{ɛ}$ is globally asymptotically stable whenever $R_{ed}<1$.

### 3.8 Existence of the disease endemic equilibrium

The endemic equilibrium point represents a steady-state solution where the disease persists in the population, defined by:

(32) $$E^{*}=\left(S_{h}^{*},A^{*},E_{h}^{*},I_{h}^{*},S_{p}^{*},E_{p}^{*},I_{p}^{*},M^{*},E^{*}\right),$$

where all state variables are strictly positive ($S_{h}^{*},A^{*},E_{h}^{*},I_{h}^{*},S_{p}^{*},E_{p}^{*},I_{p}^{*},M^{*},E^{*}>0$). This equilibrium is obtained by setting the time derivatives on the left-hand side of the model system (2) to zero.

Due to the algebraic complexity of system (2), the equilibrium state variables are expressed in terms of the equilibrium forces of infection, $\lambda_{h}^{*}$ and $\lambda_{p}^{*}$. Through systematic substitution, the coordinates of the endemic equilibrium $E^{*}$ are derived as follows:

(33) $$S_{h}^{*}=\frac{\Lambda_{h}Q_{2}}{Q_{1}Q_{2}-ɛ\rho -\frac{\gamma \theta_{h}\lambda_{h}^{*}}{D_{2}D_{3}}},$$

(34) $$A^{*}=\frac{ɛS_{h}^{*}}{\rho +\mu_{h}+\alpha \lambda_{h}^{*}},\;E_{h}^{*}=\frac{\lambda_{h}^{*}\left(S_{h}^{*}+\alpha A^{*}\right)}{D_{2}},$$

(35) $$I_{h}^{*}=\frac{\theta_{h}\lambda_{h}^{*}\left(S_{h}^{*}+\alpha A^{*}\right)}{D_{2}D_{3}},$$

(36) $$S_{p}^{*}=\frac{\Lambda_{p}}{\lambda_{p}^{*}+\mu_{p}},\;E_{p}^{*}=\frac{\lambda_{p}^{*}S_{p}^{*}}{D_{4}},$$

(37) $$I_{p}^{*}=\frac{\theta_{p}\lambda_{p}^{*}S_{p}^{*}}{D_{4}D_{5}},$$

(38) $$E^{*}=\frac{\sigma_{h}I_{h}^{*}}{\mu_{e}},\;\text{and}\;M^{*}=\frac{\sigma_{p}I_{p}^{*}}{\mu_{m}},$$

where the auxiliary variables and forces of infection at steady state are given by:

$$Q_{1}=\lambda_{h}^{*}+ɛ+\mu_{h},\;Q_{2}=\rho +\mu_{h}+\alpha \lambda_{h}^{*},$$

(39) $$\lambda_{h}^{*}=\beta_{h}M^{*},\;\text{and}\;\lambda_{p}^{*}=\beta_{p}E^{*}.$$

By substituting the expressions for the infectious classes (35), (37) into the environmental Eqs. (38), we derive explicit forms for meat contamination ($M^{*}$) and environmental egg density ($E^{*}$).

After collecting terms and simplifying, the existence of the endemic equilibrium is governed by the following quadratic polynomial in $\lambda_{h}^{*}$:

(40) $$f\left(\lambda_{h}^{*}\right)=A{\left(\lambda_{h}^{*}\right)}^{2}+B\lambda_{h}^{*}+C=0,$$

with coefficients defined as:

(41) $$A=\alpha \Upsilon \Delta +\alpha \gamma \theta_{h}\beta_{p}\Lambda_{p}\sigma_{h}\sigma_{p}\theta_{p}\cdot \frac{1}{\mu_{p}},$$

(42) $$B=\Upsilon \mu_{h}\Delta +\Upsilon \alpha \Lambda_{h}\left(1-R_{ed}^{2}\cdot \frac{\rho +\mu_{h}}{\alpha ɛ+\left(\rho +\mu_{h}\right)}\right),$$

(43) $$C=D_{2}D_{3}D_{4}D_{5}\mu_{m}\mu_{e}\mu_{h}\left(ɛ+\rho +\mu_{h}\right)\left(1-R_{ed}^{2}\right),$$

where $\Upsilon =D_{2}D_{3}D_{4}D_{5}\mu_{m}\mu_{e}$ and $\Delta =ɛ+\rho +\mu_{h}$.

Based on the derived coefficients, we establish the following analytical properties:

(i) Since all biological parameters are strictly positive, the leading coefficient satisfies A>0 unconditionally.
(ii) If $R_{ed}>1$, then $\left(1-R_{ed}^{2}\right)<0$, which implies C<0. Conversely, when $R_{ed}<1$, we have C>0.
(iii) By Descartes’ Rule of Signs (Anderson et al., 1998), a quadratic equation with A>0 and C<0 possesses exactly two real roots of opposite signs. This guarantees the existence of a unique, strictly positive root for the equilibrium force of infection, $\lambda_{h}^{*}$.
(iv) When $R_{ed}<1$, both A>0 and C>0. The sign change of C at the threshold $R_{ed}=1$ ensures that no biologically meaningful (positive) infected steady state exists when $R_{ed}<1$.

Consequently, the positivity of A combined with C<0 guarantees that the endemic equilibrium is both unique and biologically feasible whenever the basic reproduction number exceeds unity. This leads to the following result:

Theorem 6 *A unique endemic equilibrium point,* $E^{*}$*, exists in the feasible region* D *if and only if* $R_{ed}>1$*.*

### 3.9 Global stability of the endemic equilibrium

Theorem 7 *The endemic equilibrium point* $E^{*}$ *is globally asymptotically stable if in the interior of the feasible region* D*, provided that* $R_{ed}>0$ *and unstable otherwise.*

Proof To establish the global asymptotic stability of the endemic equilibrium $E_{II}^{*}$, we construct a non-linear Lyapunov function following the approach in Trazias et al. (2024):

(44) $$L^{*}=\left(S_{h}-S_{h}^{*}-S_{h}^{*}\ln\frac{S_{h}}{S_{h}^{*}}\right)+\left(A-A^{*}-A^{*}\ln\frac{A}{A^{*}}\right)+\left(E_{h}-E_{h}^{*}-E_{h}^{*}\ln\frac{E_{h}}{E_{h}^{*}}\right)+\left(I_{h}-I_{h}^{*}-I_{h}^{*}\ln\frac{I_{h}}{I_{h}^{*}}\right)+\left(S_{p}-S_{p}^{*}-S_{p}^{*}\ln\frac{S_{p}}{S_{p}^{*}}\right)+\left(E_{p}-E_{p}^{*}-E_{p}^{*}\ln\frac{E_{p}}{E_{p}^{*}}\right)+\left(I_{p}-I_{p}^{*}-I_{p}^{*}\ln\frac{I_{p}}{I_{p}^{*}}\right)+\left(E-E^{*}-E^{*}\ln\frac{E}{E^{*}}\right)+\left(M-M^{*}-M^{*}\ln\frac{M}{M^{*}}\right).$$

Differentiating Eq. (44) along time t yields

(45) $$\frac{dL^{*}}{dt}=\left(\frac{S_{h}-S_{h}^{*}}{S_{h}}\right)\left[\Lambda_{h}+\gamma_{h}I_{h}+\rho A-\left(\beta_{h}M+D_{1}\right)S_{h}\right]+\left(\frac{A-A^{*}}{A}\right)\left[ɛS_{h}-\left(\alpha \beta_{h}M+\rho +\mu_{h}\right)A\right]+\left(\frac{E_{h}-E_{h}^{*}}{E_{h}}\right)\left[\beta_{h}MS_{h}+\alpha \beta_{h}MA-D_{2}E_{h}\right]+\left(\frac{I_{h}-I_{h}^{*}}{I_{h}}\right)\left[\theta_{h}E_{h}-D_{3}I_{h}\right]+\left(\frac{S_{p}-S_{p}^{*}}{S_{p}}\right)\left[\Lambda_{p}-\left(\beta_{p}E+\mu_{p}\right)S_{p}\right]+\left(\frac{E_{p}-E_{p}^{*}}{E_{p}}\right)\left[\beta_{p}ES_{p}-D_{4}E_{p}\right]+\left(\frac{I_{p}-I_{p}^{*}}{I_{p}}\right)\left[\theta_{p}E_{p}-D_{5}I_{p}\right]+\left(\frac{E-E^{*}}{E}\right)\left[\sigma_{h}I_{h}-\mu_{e}E\right]+\left(\frac{M-M^{*}}{M}\right)\left[\sigma_{p}I_{p}-\mu_{m}M\right].$$

At the equilibrium of system (2), the following results are obtained:

(46) $$\left(\begin{array}{cc} \Lambda_{h} & =D_{1}S_{h}^{*}+\beta_{h}M^{*}S_{h}^{*}-\gamma_{h}I_{h}^{*}-\rho A^{*},\\ ɛ & =\frac{\alpha \beta_{h}M^{*}A^{*}+\rho A^{*}+\mu_{h}A^{*}}{S_{h}^{*}},\\ D_{2} & =\frac{\beta_{h}M^{*}S_{h}^{*}+\alpha \beta_{h}M^{*}A^{*}}{E_{h}^{*}},\;\;D_{3}=\frac{\theta_{h}E_{h}^{*}}{I_{h}^{*}},\\ \Lambda_{p} & =\beta_{p}S_{p}^{*}E^{*}+\mu_{p}S_{p}^{*},\;\;D_{4}=\frac{\beta_{p}S_{p}^{*}E^{*}}{E_{p}^{*}},\\ D_{5} & =\frac{\theta_{p}E_{p}^{*}}{I_{p}^{*}},\;\;\mu_{m}=\frac{\sigma_{p}I_{p}^{*}}{M^{*}},\;\;\mu_{e}=\frac{\sigma_{h}I_{h}^{*}}{E^{*}}, \end{array}\right\}$$

By substituting Eq. (46) in the Lyapunov function’s first derivative in Eq. (45), and upon simplification, yield the following results:

(47) $$\frac{dL^{*}}{dt}=\left(1-\frac{S_{h}^{*}}{S_{h}}\right)\left[\beta_{h}\left(M^{*}S_{h}^{*}-MS_{h}\right)+D_{1}\left(S_{h}^{*}+S_{h}\right)+\gamma_{h}\left(I_{h}^{*}-I_{h}\right)+\rho \left(A^{*}-A\right)\right]+\left(1-\frac{A^{*}}{A}\right)\left[\alpha \beta_{h}\left(\frac{S_{h}}{S_{h}^{*}}M^{*}A^{*}-MA\right)+\mu_{h}\left(\frac{S_{h}}{S_{h}^{*}}A^{*}-A\right)\right]+\left(1-\frac{E_{h}^{*}}{E_{h}}\right)\left[\beta_{h}M\left(S_{h}+\alpha A\right)-\beta_{h}M^{*}\left(S_{h}^{*}+\alpha A^{*}\right)\frac{E_{h}}{E_{h}^{*}}\right]+\left(1-\frac{I_{h}^{*}}{I_{h}}\right)\theta_{h}\left[E_{h}-E_{h}^{*}\frac{I_{h}}{I_{h}^{*}}\right]+\left(1-\frac{S_{p}^{*}}{S_{p}}\right)\left[\beta_{p}\left(E^{*}S_{p}^{*}-ES_{p}\right)+\mu_{p}\left(S_{p}^{*}-S_{p}\right)\right]+\left(1-\frac{E_{p}^{*}}{E_{p}}\right)\beta_{p}\left[ES_{p}-E^{*}S_{p}^{*}\frac{E_{p}}{E_{p}^{*}}\right]+\left(1-\frac{I_{p}^{*}}{I_{p}}\right)\theta_{p}\left[E_{p}-E_{p}^{*}\frac{I_{p}}{I_{p}^{*}}\right]+\left(1-\frac{E^{*}}{E}\right)\sigma_{h}\left[I_{h}-I_{h}^{*}\frac{E}{E^{*}}\right]+\left(1-\frac{M^{*}}{M}\right)\sigma_{p}\left[I_{p}-I_{p}^{*}\frac{M}{M^{*}}\right].$$

Let:

$$y_{1}=\frac{S_{h}}{S_{h}^{*}},\;y_{2}=\frac{A}{A^{*}},\;y_{3}=\frac{E_{h}}{E_{h}^{*}},\;y_{4}=\frac{I_{h}}{I_{h}^{*}},\;y_{5}=\frac{S_{p}}{S_{p}^{*}},\;y_{6}=\frac{E_{p}}{E_{p}^{*}},\;y_{7}=\frac{I_{p}}{I_{p}^{*}},y_{8}=\frac{M}{M^{*}},\;y_{9}=\frac{E}{E^{*}}.$$

Then, obtain:

(48) $$\frac{dL^{*}}{dt}=-D_{1}\frac{{\left(S_{h}-S_{h}^{*}\right)}^{2}}{S_{h}}-\mu_{p}\frac{{\left(S_{p}-S_{p}^{*}\right)}^{2}}{S_{p}}+\beta_{h}M^{*}S_{h}^{*}\left(1+y_{8}-y_{1}y_{8}-\frac{1}{y_{1}}\right)+\gamma_{h}I_{h}^{*}\left(1-y_{4}-\frac{1}{y_{1}}-\frac{y_{4}}{y_{1}}\right)+\rho A^{*}\left(1-y_{2}-\frac{1}{y_{2}}+\frac{y_{2}}{y_{1}}\right)+\beta_{h}M^{*}S_{h}^{*}\left(1+y_{1}y_{8}-y_{3}-\frac{y_{1}y_{8}}{y_{3}}\right)+\alpha \beta_{h}M^{*}A^{*}\left(y_{1}-y_{8}y_{2}-\frac{y_{1}}{y_{2}}-y_{8}\right)+\alpha \beta_{h}M^{*}A^{*}\left(1+y_{8}y_{2}-\frac{y_{8}y_{2}}{y_{3}}-y_{3}\right)+\mu_{h}A^{*}\left(y_{1}-y_{2}-\frac{y_{1}}{y_{2}}\right)+\theta_{h}E_{h}^{*}\left(1+y_{3}-y_{4}-\frac{y_{3}}{y_{4}}\right)+\beta_{p}E^{*}S_{p}^{*}\left(1+y_{9}-y_{9}y_{5}-\frac{1}{y_{5}}\right)+\beta_{p}E^{*}S_{p}^{*}\left(1+y_{9}y_{5}-y_{6}-\frac{y_{9}y_{5}}{y_{6}}\right)+\theta_{p}E_{p}^{*}\left(1+y_{6}-y_{7}-\frac{y_{6}}{y_{7}}\right)+\sigma_{p}I_{p}^{*}\left(1+y_{7}-y_{8}-\frac{y_{7}}{y_{8}}\right)+\sigma_{h}I_{h}^{*}\left(1+y_{4}-y_{9}-\frac{y_{4}}{y_{9}}\right).$$

To complete the proof of global stability, it must be shown that $\frac{dL^{*}}{dt}\leq 0$ for all points in the feasible region. This is achieved by applying the following property:

Definition 8 **Arithmetic–Geometric Mean Inequality**

For any x>0, the function g(x)=1−x+ln(x)≤0. Consequently, the inequality 1−x≤−ln(x) holds, with equality if and only if x=1 (Hardy et al., 1952).

Applying Definition 8 to the terms in our Lyapunov derivative, it follows that:

(49) $$1+y_{11}-y_{11}y_{1}-\frac{1}{y_{1}}=\left(1-y_{11}y_{1}\right)+\left(1-\frac{1}{y_{1}}\right)-\left(1-y_{11}\right)\leq -\ln\left(y_{11}y_{1}\right)-\ln\left(\frac{1}{y_{1}}\right)+\ln\left(y_{11}\right)=\ln\left(\frac{1}{y_{11}y_{1}}.y_{1}y_{11}\right)=0.$$

In a similar fashion, it follows that:

$$\left(1+y_{1}y_{8}-y_{3}-\frac{y_{1}y_{8}}{y_{3}}\right)\leq 0,\left(1-y_{2}-\frac{1}{y_{2}}+\frac{y_{2}}{y_{1}}\right)\leq 0,$$

$$\left(1-y_{4}-\frac{1}{y_{1}}-\frac{y_{4}}{y_{1}}\right)\leq 0,\left(y_{1}-y_{8}y_{2}-\frac{y_{1}}{y_{2}}-y_{8}\right)\leq 0,$$

$$\left(1+y_{8}y_{2}-\frac{y_{8}y_{2}}{y_{3}}-y_{3}\right)\leq 0,\left(y_{1}-y_{2}-\frac{y_{1}}{y_{2}}\right)\leq 0,$$

$$\left(1+y_{3}-y_{4}-\frac{y_{3}}{y_{4}}\right)\leq 0,\left(1+y_{9}-y_{9}y_{5}-\frac{1}{y_{5}}\right)\leq 0,$$

$$\left(1+y_{9}y_{5}-y_{6}-\frac{y_{9}y_{5}}{y_{6}}\right)\leq 0,\left(1+y_{6}-y_{7}-\frac{y_{6}}{y_{7}}\right)\leq 0,$$

(50) $$\left(1+y_{7}-y_{8}-\frac{y_{7}}{y_{8}}\right)\leq 0,\left(1+y_{4}-y_{9}-\frac{y_{4}}{y_{9}}\right)\leq 0.$$

Hence:

$$\frac{dL^{*}}{dt}\leq -D_{1}\frac{{\left(S_{h}-S_{h}^{*}\right)}^{2}}{S_{h}}-\mu_{p}\frac{{\left(S_{p}-S_{p}^{*}\right)}^{2}}{S_{p}},\;\;\;\frac{dL^{*}}{dt}\leq 0.$$

It has been shown that $\frac{dL^{*}}{dt}\leq 0$ within the feasible region D, with $\frac{dL^{*}}{dt}=0$ holding if and only if the system is at the endemic equilibrium $E^{*}$. Consequently, the singleton $E^{*}$ is the largest invariant set in D where the derivative of the Lyapunov function vanishes. According to LaSalle’s Invariance Principle (LaSalle, 1976), every solution of the model (2) starting in D approaches $E^{*}$ as t→∞, provided that $R_{ed}>1$. Therefore, the endemic equilibrium $E^{*}$ is globally asymptotically stable in D for $R_{ed}>1$.

### 3.10 Bifurcation behaviour

To investigate the topological nature of the system’s equilibria transitions, we analyse the bifurcation behaviour at the critical threshold $R_{ed}=1$. By virtue of the global asymptotic stability established for the disease-free equilibrium $E_{ɛ}$ in Section 3.6, the system strictly rules out the phenomenon of a subcritical (backward) bifurcation, as no stable endemic equilibria can co-exist within the basin of attraction when $R_{ed}<1$.

Consequently, the model undergoes a classic *forward (supercritical) bifurcation* at $R_{ed}=1$. As $R_{ed}$ increases past unity, the disease-free equilibrium loses its stability, and a unique, locally stable endemic equilibrium branches out into the feasible domain D. From an epidemiological control standpoint, this forward bifurcation structure confirms that lowering the educational transmission threshold below unity ($R_{ed}<1$) is both a necessary and entirely sufficient condition to guarantee the absolute eradication of Taeniasis from the population.

## 4 Numerical results and simulations

In this section, we present numerical simulations to validate the theoretical findings derived in the preceding analytical sections. The investigation is structured into three primary components: a comprehensive sensitivity analysis of the effective reproduction number ($R_{ed}$), an evaluation of the impact of educational parameters on disease threshold dynamics, and a trajectory analysis of the global endemic regime using the baseline parameters detailed in Table 1. To assess the efficacy of behavioural interventions, we explore the parameter space of the education-related variables (ɛ,α, and ρ), illustrating how shifts in public health dissemination and knowledge retention drive the dynamical transition between disease persistence and elimination.

### 4.1 Sensitivity analysis for the reproduction number ($R_{ed}$)

Following the rigorous stability analysis of the system, this section investigates the sensitivity of the control reproduction number, $R_{ed}$, to the parameters specifically associated with the education intervention. The primary objective of this sensitivity analysis is to quantify the relative impact of educational strategies on the transmission and persistence of *T. solium*. By identifying the most influential parameters — such as the education rate α or the efficacy of behavioural change ɛ — we can determine which interventions are most effective in shifting the system from an endemic state towards disease eradication.

Following the approach described by Chitnis et al. (2008), we employ the normalized forward sensitivity index (also referred to as elasticity) to evaluate the relative importance of different parameters on the control reproduction number, $R_{ed}$. Given that $R_{ed}$ is differentiable with respect to a given parameter p, the sensitivity index $\Upsilon_{p}^{R_{ed}}$ is defined as the ratio of the relative change in $R_{ed}$ to the relative change in p:

(51) $$\Upsilon_{p}^{Red}=\frac{\partial Red}{\partial p}\times \frac{p}{R_{ed}}.$$

By applying the formula (51) to each parameter within the expression for $R_{ed}$, we derive the following analytical results (Table 2). These indices represent the elasticity of the control reproduction number with respect to each parameter, providing a quantitative basis for prioritizing interventions.

The sensitivity indices presented in Table 2 indicate that parameters with negative values are inversely proportional to $R_{ed}$. Specifically, an increase in these parameters results in a decrease in $R_{ed}$, identifying them as critical targets for disease burden reduction. Conversely, parameters with positive indices show a direct proportional relationship, suggesting that a reduction in these values effectively lowers the transmission potential.

**Table 2:** Normalized sensitivity indices of $R_{ed}$ for non-demographic parameters, illustrating the relative impact of transmission and intervention factors.

**Parameter**	$\beta_{h}$	$\beta_{p}$	$\sigma_{h}$	$\sigma_{p}$	ρ	
**Index**	+0.5000	+0.5000	+0.5000	+0.5000	+0.5000	

**Parameter**	$\theta_{h}$	$\theta_{p}$	$\mu_{m}$	$\mu_{e}$	α	ɛ
**Index**	+0.5000	+0.5000	−0.5000	−0.5000	−0.3421	−0.3421

**Fig. 2:**
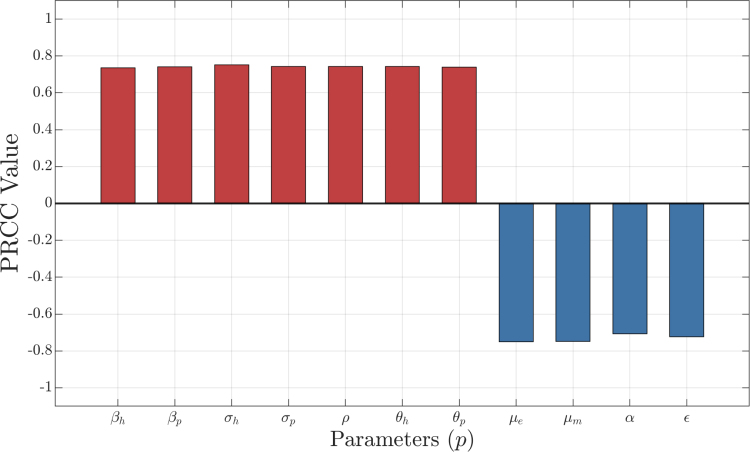
Partial Rank Correlation Coefficients (PRCC) for the education-dependent reproduction number $R_{ed}$.

To account for the inherent uncertainty and simultaneous variation in model parameters, the local sensitivity analysis is complemented by a global sensitivity analysis. Utilizing the Latin Hypercube Sampling (LHS) scheme in conjunction with Partial Rank Correlation Coefficients (PRCC), the results are illustrated in Fig. 2. The analysis quantifies the global sensitivity of the control reproduction number, $R_{ed}$, across the entire parameter space.

Mathematically, the LHS scheme operates by dividing the probability distribution of each parameter $P_{j}$ (for j=1,2,…,k) into N non-overlapping intervals of equal probability 1/N (McKay et al., 1979). For the ith sampling iteration, the parameter value is selected according to:

(52) $$P_{ji}=F_{j}^{-1}\left(\frac{\pi_{j}\left(i\right)-U_{ji}}{N}\right)$$

where $F_{j}^{-1}$ is the inverse cumulative distribution function of parameter $P_{j}$, $\pi_{j}$ is a random permutation of the integers {1,2,…,N}, and $U_{ji}$ is a uniform random variable on [0,1). This guarantees a thorough exploration of the multi-dimensional parameter space.

The PRCC values are then calculated to evaluate the sensitivity of the target threshold, $R_{ed}$, to each parameter following the methodology outlined by (Marino et al., 2008). Let $x_{j}$ denote the rank-transformed data of parameter $P_{j}$, and let y denote the rank-transformed data of $R_{ed}$. To eliminate the linear influences of all other simultaneously varying parameters, we define linear regression models for both $x_{j}$ and y with respect to the remaining parameters:

(53) $${\overset{ˆ}{x}}_{j}=c_{0}+\underset{m\neq j}{\sum }c_{m}x_{m},\;\text{and}\;\overset{ˆ}{y}=b_{0}+\underset{m\neq j}{\sum }b_{m}x_{m}$$

The residual vectors, which capture the unexplained variance, are given by ${\overset{\sim }{x}}_{j}=x_{j}-{\overset{ˆ}{x}}_{j}$ and $\overset{\sim }{y}=y-\overset{ˆ}{y}$. The PRCC between $P_{j}$ and $R_{ed}$ is defined as the standard Pearson correlation coefficient between these residuals:

(54) $$\text{PRCC}\left(P_{j},R_{ed}\right)=\frac{\overset{N}{\underset{i=1}{\sum }}{\overset{\sim }{x}}_{ji}{\overset{\sim }{y}}_{i}}{\sqrt{\overset{N}{\underset{i=1}{\sum }}{\overset{\sim }{x}}_{ji}^{2}}\sqrt{\overset{N}{\underset{i=1}{\sum }}{\overset{\sim }{y}}_{i}^{2}}}$$

The resulting values lie within [−1,1], where the sign indicates the direction of the impact and the magnitude quantifies the sensitivity.

As shown in the PRCC results (Fig. 2), the transmission coefficients $\beta_{h}$ and $\beta_{p}$ significantly drive the epidemic, as their reduction directly lowers infection rates. For instance, a 100% reduction in human exposure — achieved through behavioural change — results in a 79% decline in infections, highlighting the importance of educational campaigns. Furthermore, the parameters $\mu_{e}$ and ɛ were found to influence infection dynamics with the same magnitude but in an inverse direction. Notably, the intervention parameters α and ϵ exhibit a significant negative correlation, and ρ positively correlated; underscoring the potent impact of education-based strategies in mitigating disease transmission.

### 4.2 Impact of education parameters on the reproduction number ($R_{ed}$)

**Fig. 3:**
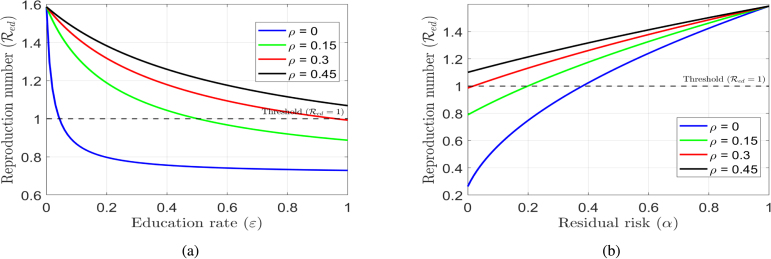
Univariate sensitivity analysis of the reproduction number $R_{ed}$ with respect to the (a) education rate ɛ, and (b) residual risk α for varying levels of knowledge decay ρ∈{0,0.15,0.30,0.45}. Other parameters are held at baseline values.

**Fig. 4:**
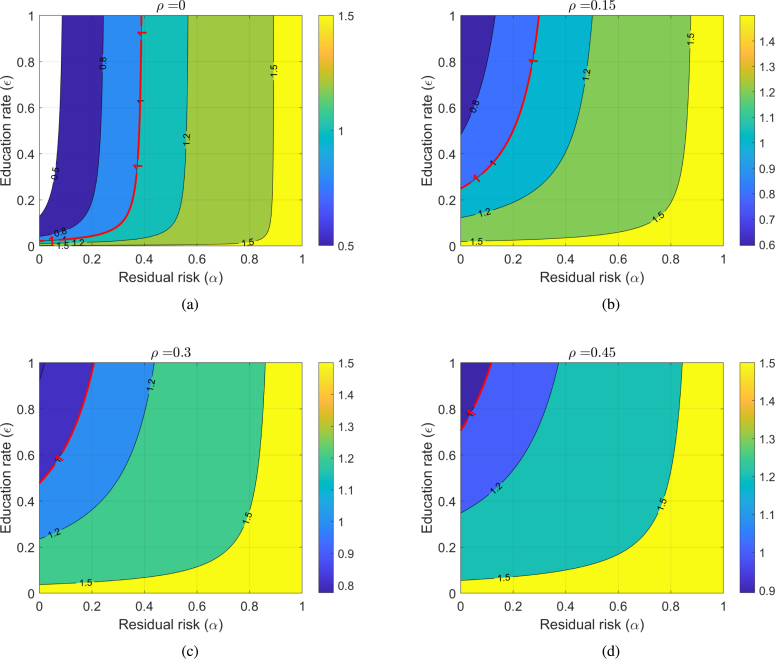
Combined 2D contour plots of the reproduction number $R_{ed}$ as a function of dissemination rate ɛ and residual risk α. The colour gradient represents the magnitude of $R_{ed}$, with the red line indicating the critical threshold $R_{ed}=1$.

In this section, we investigate the influence of three key education-related parameters: the rate of education dissemination (ɛ), the residual risk or strategy effectiveness (α), and the knowledge retention rate (ρ), which accounts for the loss of awareness within the educated population. We first conduct a univariate sensitivity analysis for ɛ and α across varying levels of ρ, followed by an examination of their combined synergistic effects on the reproduction number ($R_{ed}$). Numerical simulations of the education-dependent reproduction number ($R_{ed}$) — defined as the expected number of secondary infections in both human and pig populations generated by a single infected individual in a community implementing the proposed interventions — are illustrated in Fig. 3, Fig. 4. These figures depict the univariate sensitivity of the reproduction number and the synergistic effects of combined parameters, respectively.

The numerical simulations presented in Fig. 3(a) illustrate the impact of varying knowledge dissemination rates (ɛ) across different levels of knowledge retention (ρ). This analysis accounts for the inherent uncertainty and variability in how effectively public health education prevents infection, reflecting real-world factors such as behavioural responses and the natural waning of awareness over time. Epidemiologically, the results mean that one infected human or pig cannot infect others whenever the stated thresholds are observed per day; otherwise, one infected human or pig can infect on average 0.6 to 1.6 humans and pigs during the entire infection period under this strategy set up.

Nevertheless, the findings demonstrate that for an education strategy to be successful, the dissemination rate must reach a critical threshold to overcome the rate of knowledge decay. Specifically, when the knowledge loss rate (ρ) is maintained at or below 0.30, achieving a disease-free state ($R_{ed}<1$) requires the education of at least 85% of the susceptible human population per day (ɛ≥0.85). As further observed in Fig. 3(a), the reproduction number exhibits a non-linear decay as the dissemination rate increases, highlighting a critical tipping point beyond which the system transitions from endemic persistence to disease elimination. Since the analysis requires a dissemination rate of 0.85, health authorities should prioritize “saturation” campaigns. This includes the use of mass media, community workshops, and school-based programmes to ensure that the vast majority of the susceptible population is reached rapidly.

The simulation for sensitivity of the reproduction number $R_{ed}$ to the effectiveness of education strategies (α) is illustrated in Fig. 3(b). The parameter α represents the ‘residual risk’—the failure of educational awareness to translate into complete preventive behaviour. It can be noticed that as α increases (indicating lower educational efficacy), $R_{ed}$ rises linearly, confirming that the quality of the intervention is a primary driver of transmission dynamics. Epidemiologically, the results mean that one infected human or pig cannot infect others whenever the stated thresholds are observed per day; otherwise, one infected human or pig can infect on average 0.6 to 1.4 humans and pigs during the entire infection period under this strategy setup.

Notably, the results show that when knowledge retention is high (ρ=0), the community can tolerate a higher residual risk (α≈0.40) while still maintaining $R_{ed}<1$. However, as knowledge decay increases to ρ=0.45, even a highly effective strategy (low α) may fail to bring the reproduction number below the elimination threshold. This suggests a synergistic vulnerability: poor educational quality compounded by rapid forgetting creates a ‘refuge’ for the parasite, allowing for endemic persistence even under active intervention.

Epidemiologically, these results imply that the chain of transmission is effectively broken — preventing a single infected human or pig from generating secondary cases — provided the identified intervention thresholds are strictly maintained. Conversely, failure to meet these critical values allows for an endemic state where an infected individual can, on average, transmit the parasite to between 0.6 and 1.4 other hosts during their infectious period. Consequently, public health interventions must evolve beyond simple knowledge dissemination towards behavioural verification. While awareness of *Taenia solium* is foundational, it is insufficient if not supported by physical infrastructure. The provision of essential resources — such as soap, potable water, and latrine maintenance kits — is mandatory to bridge the gap between knowledge and practice, thereby minimizing the residual risk (α). Furthermore, to optimize educational efficacy, materials must be culturally tailored and linguistically accessible; generic health communication often yields a high α due to interpretative misalignment. Therefore, institutional funding should prioritize local health ambassadors capable of providing context-specific, hands-on demonstrations to ensure high-fidelity behavioural change

Complementing the univariate analysis, the results presented in Fig. 4 illustrate the transmission potential of *T. solium* when the dissemination rate (ɛ) and the quality of educational materials — quantified by the residual risk (α) — are varied simultaneously. These bifactorial simulations are conducted across various scenarios of knowledge retention (ρ) to evaluate how the interplay between educational reach and efficacy determines the feasibility of disease elimination.

As observed across the four scenarios in Figs. 4(a)–(d), a rise in the knowledge decay rate (ρ) causes the disease elimination region ($R_{ed}<1$) to shrink significantly. Under the ideal condition of perfect knowledge retention (ρ=0), a broad spectrum of dissemination rates (ɛ) and residual risk levels (α) facilitates disease control. However, as the decay rate increases to 45%, the required dissemination rate must exceed 0.9, even when the residual risk is minimized. These findings reveal that high-speed dissemination alone cannot compensate for rapid knowledge loss; rather, a sustainable eradication strategy necessitates an integrated approach that simultaneously accelerates information spread while actively slowing the rate of forgetting. Furthermore, since $R_{ed}$ is governed by the dynamics of both host populations, educational interventions must emphasize the critical link between porcine management and human health. Such an approach ensures that the community understands that human behavioural change is only one half of the solution, while the management of the intermediate pig host completes the eradication effort. Consequently, public health policies should incentivize the construction of sanitary latrines and the strict confinement of pigs to effectively disrupt the transmission cycle.

### 4.3 Numerical dynamical simulations

**Fig. 5:**
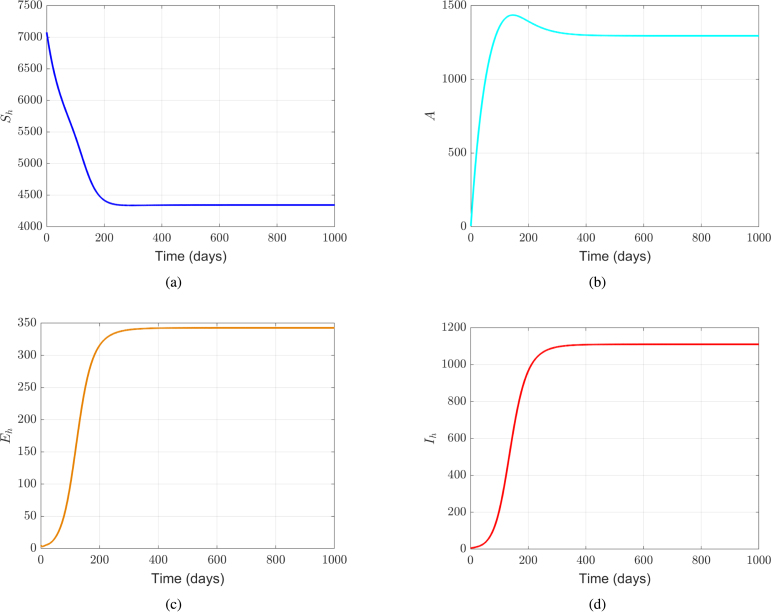
Temporal dynamics of the human compartments in model system (2) under the endemic scenario $R_{ed}=2.123>1$. The panels depict the populations of (a) susceptible individuals, (b) educated individuals, (c) exposed individuals, and (d) infected individuals. Simulations are based on the parameter values in Table 1, with the education dissemination rate $ɛ=4.31\times 10^{-3}$ and the residual risk α=0.03.

**Fig. 6:**
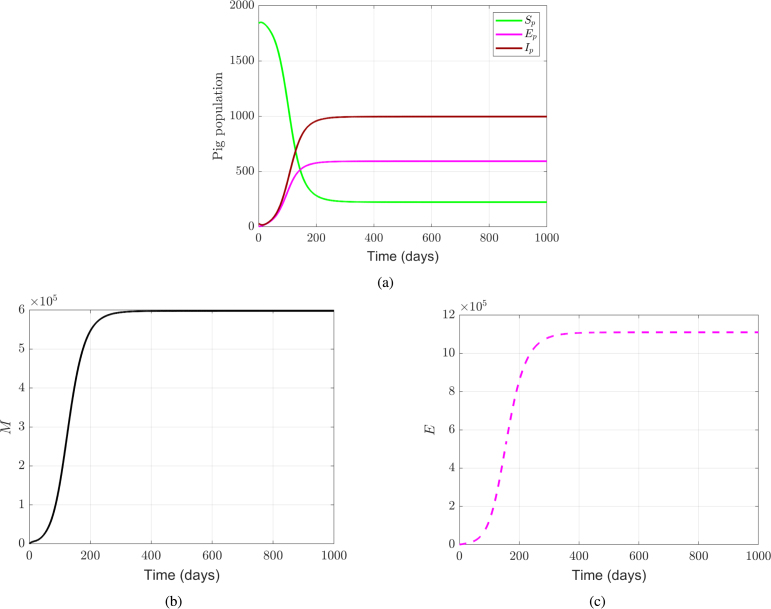
Temporal dynamics of the pig and environmental compartments in model system (2) under the endemic scenario $R_{ed}=2.123>1$. The panels illustrate the trajectories for (a) the infected pig population, (b) the concentration of *T. solium* larvae in the environment, and (c) the abundance of *T. solium* eggs. Simulations utilize parameter values from Table 1 with an education dissemination rate $ɛ=4.31\times 10^{-3}$ and a residual risk factor α=0.03.

To illustrate the theoretical results, numerical simulations of the mathematical model (2) are performed using baseline parameter values as presented in Table 1 with initial conditions are set as: $S_{h}\left(0\right)=10\;000,A\left(0\right)=5,E_{h}\left(0\right)=5,I_{h}\left(0\right)=5,S_{p}\left(0\right)=2\;000,E_{p}\left(0\right)=5,I_{p}\left(0\right)=30,M\left(0\right)=1000$, and E(0)=1000. The simulation results, depicting the population dynamics and infection trajectories, are presented in Fig. 5, Fig. 6.

The numerical simulation in Fig. 5(a) illustrates the temporal dynamics of the susceptible human compartment over a 1000-day period. The population exhibits a gradual decline before reaching a steady-state convergence at a non-zero equilibrium after approximately 300 days. This initial reduction is driven by three primary factors: first, the transition of individuals into the educated (aware) class through the dissemination of public health information (ɛ); second, the contraction of Taeniasis via the consumption of infected pork; and third, the natural mortality rate ($\mu_{h}$) applied across all human compartments. Crucially, the stabilization of the susceptible population at a non-zero level, rather than its total depletion, highlights the impact of educational interventions. This indicates that the community maintains a resilient susceptible pool protected by awareness, preventing the entire population from remaining at risk of infection.

The dynamics of the educated (aware) class, $A_{h}$, are illustrated in Fig. 5(b). This compartment is populated by individuals from the susceptible class, $S_{h}$, following the successful dissemination of public health information. The numerical results show a rapid increase in the educated population within the first 100 days, reaching a prominent peak before undergoing a gradual decline and eventually stabilizing at a steady state around the 400th day. This observed decline following the peak is attributed to the interplay of three factors: the attrition of knowledge (ρ), where individuals revert to the susceptible class; the transition of aware individuals into the exposed class ($E_{h}$) due to residual transmission risk (α); and the natural mortality rate ($\mu_{h}$). The substantial initial growth in the number of educated individuals reflects the high efficacy of early-stage dissemination and the continuous recruitment of susceptible individuals into the awareness program.

Figs. 5(c) and (d) illustrate the temporal evolution of the exposed ($E_{h}$) and infected ($I_{h}$) human populations, respectively, under an environment moderated by educational interventions. Both compartments exhibit a gradual growth phase, reflecting the latent period of infection and the transmission dynamics of the parasite. Following this initial expansion, both populations reach a peak magnitude before converging to a stable endemic equilibrium after approximately 400 days. This stabilization at a non-zero level confirms that, under the current parameter set where $R_{ed}>1$, the disease persists in the population despite the intervention. However, the relatively low steady-state density of infected individuals — compared to the susceptible pool — underscores the role of education in suppressing the overall burden of Taeniasis, even when eradication thresholds are not fully met. Ultimately, the convergence to a stable, non-zero equilibrium highlights the long-term sustainability of the educational intervention within the population.

A consistent trend is observed in the pig population, as illustrated in Fig. 6(a), reflecting the indirect impact of human-targeted educational campaigns on the porcine transmission cycle. The dynamics captured in this period represent an endemic state, occurring because the educational intervention — while present — is insufficient to drive the effective reproduction number ($R_{ed}$) below the eradication threshold. As shown in Fig. 6(a), the susceptible pig population undergoes a rapid decline during the first 400 days as individuals transition into the infected classes. However, due to the constant recruitment of new susceptible pigs ($\Lambda_{p}$), the population does not deplete to zero but instead stabilizes at a non-zero steady state. Concurrently, as a significant portion of the susceptible pool acquires the infection from the contaminated environment (eggs), the numbers of exposed and infected pigs exhibit a sharp increase during the initial 400 days before reaching their respective endemic equilibria. This stabilization underscores the persistence of the parasite within the intermediate host, fuelled by the environmental reservoir despite the human behavioural interventions

Figs. 6(b) and (c) demonstrate the concentrations of *T. solium* eggs and larvae in contaminated environments and pork meat products, respectively. During the first 400 days, both concentrations exhibit a sharp increase before undergoing a gradual decline and stabilizing at a non-zero steady state. This initial surge in the environmental egg concentration is primarily driven by the shedding rate of eggs from infected humans ($I_{h}$) into the environment. Simultaneously, the recruitment of infected pigs into the slaughterhouse or market system — represented by the harvesting rate — contributes to the rapid rise in *T. solium* larval concentration within available pork products. The stabilization of the aforementioned concentrations after approximately 400 days highlights the transition from the transient outbreak phase to a sustained endemic state. Therefore, these findings underscore the environmental and dietary pathways that maintain the parasite’s life cycle, emphasizing that as long as $R_{ed}>1$, a persistent reservoir of eggs and larvae will exist, posing a continuous reinfection risk to both human and porcine hosts.

### 4.4 Impact of public health education on infection dynamics

**Fig. 7:**
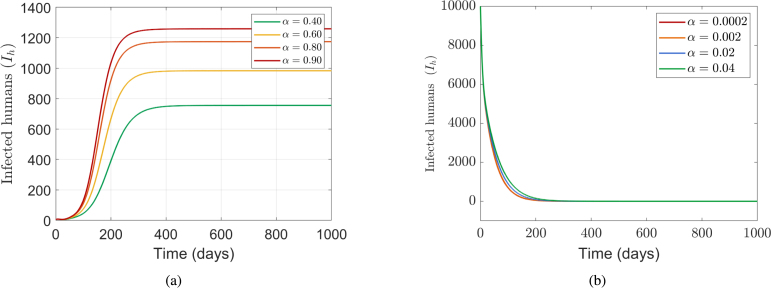
Impact of varying the residual risk parameter α on the temporal dynamics of Taeniasis-infected individuals. Panel (a) illustrates the endemic state where $R_{ed}>1$, using α={0.4,0.6,0.8,0.9}. Panel (b) depicts the disease eradication phase where $R_{ed}<1$, using α={0.0002,0.002,0.02,0.04,1.0}. Simulations are conducted with fixed intervention parameters ρ=0.00005 and ɛ=0.08; all other parameter values are as specified in Table 1.

This section explores the effectiveness of educational interventions by varying the risk-reduction parameter α within the human compartments. Fig. 7(a) examines the influence of α on the total density of individuals with Taeniasis under the condition $R_{ed}>1$. The results indicate that minimizing the value of α (representing high educational efficacy) significantly suppresses the endemic steady state, whereas higher values of α allow for the continued persistence of infection. Conversely, Fig. 7(b) illustrates the dynamics when $R_{ed}<1$. The numerical analysis reveals that as α approaches zero, the system converges more rapidly towards the disease-free equilibrium, effectively leading to the extinction of the pathogen within the population.

The findings in Fig. 7 suggest that when $R_{ed}>1$, public health education alone significantly reduces the final density of *T. solium* infections, even if it does not independently drive the reproduction number below the eradication threshold. Our results show that lower values of α lead to a marked decrease in disease prevalence. Consequently, we recommend a multi-modal strategy where intensified education — aimed at minimizing α — is paired with additional measures such as mass drug administration or porcine vaccination. This supports our theoretical analysis, which demonstrates that lowering the residual risk α through public health education is a vital component in reducing the model’s education dependent reproduction number, $R_{ed}$.

### 4.5 Synergistic impact of education rate (ɛ) and efficacy (α)

**Fig. 8:**
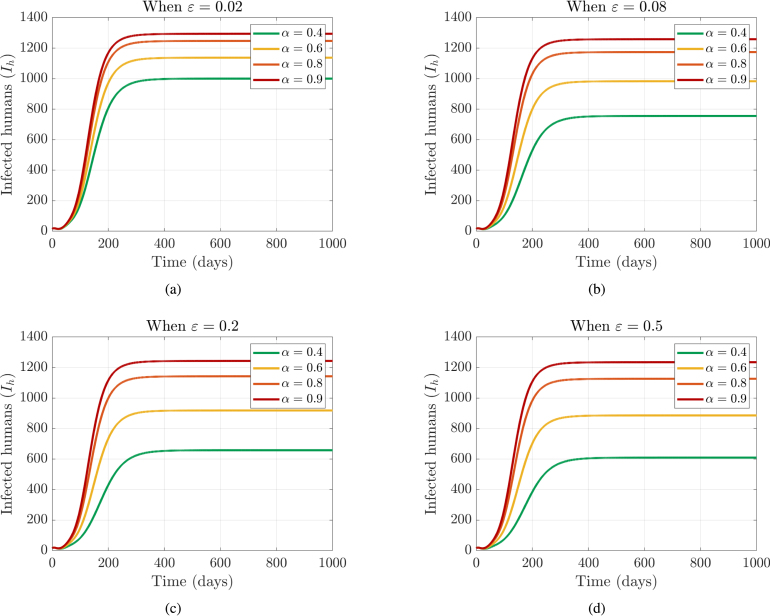
Dynamic response of the infected human population ($I_{h}$) to variations in educational efficacy (α) under fixed dissemination rates (ɛ). Panels (a)–(d) illustrate how increasing the dissemination speed from low to high enhances the impact of risk-reduction (α), where lower α values represent higher compliance and effectiveness. Baseline parameters are as specified in Table 1.

This section investigates the joint influence of the education dissemination rate (ɛ) and the risk-reduction efficacy (α) on the temporal dynamics of the infected human population ($I_{h}$). To evaluate the impact of behavioural change quality, the parameter α is varied across a spectrum of residual risk levels (α∈{0.02,0.08,0.2,0.5}), while the reach of the intervention is examined at various constant dissemination rates (ɛ∈{0.4,0.6,0.8,0.9}). Utilizing the baseline parameter estimates provided in Eq. (1), the model (2) was numerically simulated to capture the outbreak trajectories. The resulting dynamics, illustrated in Fig. 8, demonstrate how the interplay between the speed of information spread and the effectiveness of subsequent behavioural shifts dictates the magnitude of the epidemic peak and the rate of convergence to the disease-free equilibrium.

The findings illustrated in Fig. 8 highlight that the total density of infected individuals is highly sensitive to the synergy between the speed of educational dissemination (ɛ) and its effectiveness in reducing transmission risk (α). For instance, Fig. 8(b) demonstrates that even high levels of efficacy are largely wasted if the information remains unreachable due to a low dissemination rate (ɛ). In contrast, Fig. 8(c) shows that moderate dissemination allows for a more pronounced impact of the efficacy parameter α on the epidemic curve. Finally, Fig. 8(d) depicts a ’fast-reach’ scenario, underscoring that optimal control is achieved when rapid outreach (increasing ɛ) is paired with high compliance (reducing α). This numerical analysis aligns with the theoretical results established in Section 4.4.

## 5 Conclusion

*Taenia solium* remains a significant threat to both public health and economic stability in endemic regions of Sub-Saharan Africa. Failure to implement robust, evidence-based interventions will likely exacerbate this burden, leading to persistent morbidity and continued livestock losses. Consequently, this study formulated and rigorously analysed a mathematical model for *T. solium* Taeniasis, incorporating public health education as a primary non-pharmaceutical intervention. By accounting for the dynamics of behavioural modification and knowledge attrition, this work provides a theoretical framework to investigate the critical role of community-based control measures in breaking the transmission cycle. In this study, both the basic reproduction number, $R_{0}^{\left(L\right)}$, and the education-influenced effective reproduction number, $R_{ed}$, were derived and compared to evaluate the transmission threshold. We formally established the existence and local stability of the disease-free equilibrium (DFE), followed by a rigorous proof of its global stability when $R_{ed}<1$. Furthermore, the existence and global asymptotic stability of the endemic equilibrium were demonstrated, ensuring that the disease persists at a unique steady state when the threshold is exceeded.

These analytical findings were validated through numerical simulations, which provide robust support for the theoretical threshold conditions. Notably, the sensitivity analysis identified the transmission rates ($\beta_{h},\beta_{p}$) and the environmental contamination parameters ($\sigma_{h},\sigma_{p}$) as the most influential factors driving the epidemic. The simulations further reveal that reaching a critical education efficacy — where the scaling factor α is significantly reduced — is sufficient to suppress $R_{ed}$ below unity. However, the long-term success of such interventions is strictly contingent upon a knowledge-attrition rate (ρ) that does not outpace the rate of education dissemination.

While our methodological framework shares foundational elements with previous mathematical studies, this work specifically elucidates the dynamics of *T. solium* under behavioural modification. The results align with and extend prior epidemiological findings, such as those reported in Nyang’inja et al., 2018, Althubyani, 2021, highlighting the necessity of sustained, iterative public health campaigns. Ultimately, the model suggests that a “One Health” approach, which prioritizes continuous community engagement alongside porcine management, remains the most viable pathway towards the elimination of *T. solium* in resource-limited settings.

Like all mathematical frameworks, the proposed model is subject to certain limitations that warrant acknowledgement. First, the model assumes homogeneous mixing between human and porcine hosts and does not account for spatial heterogeneity in exposure risk, which is often a critical factor in rural transmission sites. Second, while education is represented as a transition to an ‘Aware’ class, behavioural change in real-world populations often occurs more gradually than the instantaneous rates modelled here. Furthermore, environmental contamination is simplified into two discrete parasite stages and does not explicitly capture the climatic or seasonal variability that may affect egg viability. Finally, the deterministic structure of this model neglects stochastic effects, which can be significant during the early stages of an outbreak or in small, isolated communities.

For future research, we propose extending this framework to incorporate spatial structuring, stochasticity, and seasonal forcing. Additionally, exploring the impact of feedback-driven awareness — where the intensity of public health education is dynamically linked to the observed prevalence of the disease — would provide deeper insights into the long-term sustainability of *T. solium* elimination strategies.

## CRediT authorship contribution statement

**Gideon Eustace Rwabona:** Writing – review & editing, Writing – original draft, Visualization, Validation, Methodology, Investigation, Formal analysis, Conceptualization. **Abdoelneser Degoot:** Writing – review & editing, Visualization, Validation, Supervision. **Silas Mirau:** Writing – review & editing, Visualization, Validation, Supervision. **Sayoki Mfinanga:** Writing – review & editing, Visualization, Validation, Supervision. **Verdiana Grace Masanja:** Writing – review & editing, Visualization, Validation, Supervision.

## Declaration of Generative AI and AI-assisted technologies in the writing process

During the preparation of this work, the authors did not use generative AI or AI-assisted technologies in the writing process.

## Funding

This work did not receive any specific funding.

## Declaration of competing interest

The authors declare that they have no known competing financial interests or personal relationships that could have appeared to influence the work reported in this paper.

## Data Availability

All data analysed during this study are included in this published article. The model parameters were derived from previously published literature and are cited accordingly within the manuscript. No new primary data were generated or analysed during the current study.
